# Altered DNA Methylation in Leukocytes with Trisomy 21

**DOI:** 10.1371/journal.pgen.1001212

**Published:** 2010-11-18

**Authors:** Kristi Kerkel, Nicole Schupf, Kota Hatta, Deborah Pang, Martha Salas, Alexander Kratz, Mark Minden, Vundavalli Murty, Warren B. Zigman, Richard P. Mayeux, Edmund C. Jenkins, Ali Torkamani, Nicholas J. Schork, Wayne Silverman, B. Anne Croy, Benjamin Tycko

**Affiliations:** 1 Institute for Cancer Genetics, Columbia University Medical Center, New York, New York, United States of America; 2 Taub Institute for Research on Alzheimer's disease and the Aging Brain, Columbia University Medical Center, New York, New York, United States of America; 3 Departments of Human Genetics, Epidemiology, and Psychiatry, Institute for Basic Research on Developmental Disabilities, New York, New York, United States of America; 4 Departments of Anatomy and Cell Biology and Microbiology and Immunology, Queen's University, Kingston, Canada; 5 Department of Pathology, Columbia University Medical Center, New York, New York, United States of America; 6 Department of Medical Oncology and Hematology and Department of Medical Biophysics, University of Toronto and Princess Margaret Hospital, Toronto, Canada; 7 Department of Neurology, Columbia University Medical Center, New York, New York, United States of America; 8 Scripps Translational Science Institute, La Jolla, California, United States of America; 9 Department of Behavioral Psychology, Kennedy Krieger Institute, Baltimore, Maryland, United States of America; 10 Department of Psychiatry and Behavioral Sciences, Johns Hopkins University School of Medicine, Baltimore, Maryland, United States of America; Friedrich Miescher Institute for Biomedical Research, Switzerland

## Abstract

The primary abnormality in Down syndrome (DS), trisomy 21, is well known; but how this chromosomal gain produces the complex DS phenotype, including immune system defects, is not well understood. We profiled DNA methylation in total peripheral blood leukocytes (PBL) and T-lymphocytes from adults with DS and normal controls and found gene-specific abnormalities of CpG methylation in DS, with many of the differentially methylated genes having known or predicted roles in lymphocyte development and function. Validation of the microarray data by bisulfite sequencing and methylation-sensitive Pyrosequencing (MS-Pyroseq) confirmed strong differences in methylation (p<0.0001) for each of 8 genes tested: *TMEM131*, *TCF7*, *CD3Z/CD247*, *SH3BP2*, *EIF4E*, *PLD6*, *SUMO3*, and *CPT1B*, in DS versus control PBL. In addition, we validated differential methylation of *NOD2/CARD15* by bisulfite sequencing in DS versus control T-cells. The differentially methylated genes were found on various autosomes, with no enrichment on chromosome 21. Differences in methylation were generally stable in a given individual, remained significant after adjusting for age, and were not due to altered cell counts. Some but not all of the differentially methylated genes showed different mean mRNA expression in DS versus control PBL; and the altered expression of 5 of these genes, *TMEM131*, *TCF7*, *CD3Z*, *NOD2*, and *NPDC1*, was recapitulated by exposing normal lymphocytes to the demethylating drug 5-aza-2′deoxycytidine (5aza-dC) plus mitogens. We conclude that altered gene-specific DNA methylation is a recurrent and functionally relevant downstream response to trisomy 21 in human cells.

## Author Summary

Down syndrome (DS; trisomy 21) is caused by the gain of a single extra chromosome 21. However, the mechanisms by which this extra chromosome produces the medical abnormalities seen in DS, including not only mental retardation but also susceptibility to autoimmune diseases and recurrent infections, are still not understood. DNA methylation is a mechanism that might contribute to these abnormalities. To test this possibility, we profiled DNA methylation in white blood cells from adults with DS and normal controls and found recurrent abnormalities of gene methylation in DS, with several of the differentially methylated genes having roles in blood cells. Among the genes with hypo- or hyper-methylation in white blood cells or purified T-lymphocytes from adults with DS, compared to these same types of cells from normal adults, were *TMEM131*, *TCF7*, *CD3Z*, *SH3BP2*, *EIF4E*, *SUMO3*, *CPT1B*, *NOD2/CARD15*, *NPDC1*, and *PLD6*. Several of these genes showed not only different methylation but also different expression in DS versus control blood cells, which was recapitulated by exposing normal white blood cells to a demethylating drug. These findings show that altered DNA methylation of a specific group of genes is a fundamental cellular response to the gain of an extra chromosome 21 in humans. The abnormally methylated genes identified here may contribute to immune system abnormalities in people with DS.

## Introduction

It is now 5 decades since Down syndrome (DS) was first shown to result from trisomy 21 [1], [2], and some progress has been made toward understanding the genes that contribute to the complex array of DS phenotypes – mostly by studying the effects of the trisomy on transcriptional profiles in humans and mice and by creating transgenic and trans-chromosomal mouse models [3], [4]. We are still far from understanding the mechanisms that underlie the complex spectrum of phenotypes in DS. Survival in DS can range from death *in utero* to late adulthood; cardiac defects are present in about 40% of cases, while cognitive disability is invariably present but can range from mild to severe. Additionally, there are multiple blood cell-related phenotypes including leukemoid reactions and childhood leukemias, macrocytosis with or without anemia, a markedly increased incidence of autoimmune disorders, and increased susceptibility to recurrent bacterial and viral infections [5]–[10].

All of these abnormalities must ultimately reflect the downstream responses of human cells and tissues to the chromosome 21 aneuploidy. In theory, one mechanism by which cells might respond to changes in gene dosage is altered DNA methylation. Gain of methylation at cytosines in CpG dinucleotides in promoter-associated CpG islands (CGI's) can enforce dosage compensation in X-inactivation, and methylation in other types of CG-rich sequences including intragenic sequences and insulator elements can affect expression and hence functional gene dosage at imprinted loci. With these simple ideas in mind we set out to ask whether gains or losses of genomic DNA methylation might occur as a downstream consequence of trisomy 21 in blood cells from adults with DS. Studies profiling mRNA expression in cells and tissues with trisomy 21 have shown that while many genes on chromosome 21 are over-expressed, subsets of genes on other chromosomes also show consistently altered expression in this background due to gene-gene interactions (for example [11]–[15]). So in testing for epigenetic changes downstream of trisomy 21 it is important to examine the whole genome. Here we show that a small group of genes, distributed across various chromosomes and not over-represented on chromosome 21, are consistently altered by recurrent gains or losses of DNA methylation in PBL of adults with DS. For a subset of these genes we find altered mRNA expression in DS versus control blood cells, and we show that this altered expression can be recapitulated by exposing normal lymphocytes to the demethylating drug 5aza-dC.

## Results

### Microarray-based profiling of DNA methylation in DS versus control PBL

**Figure 1:**
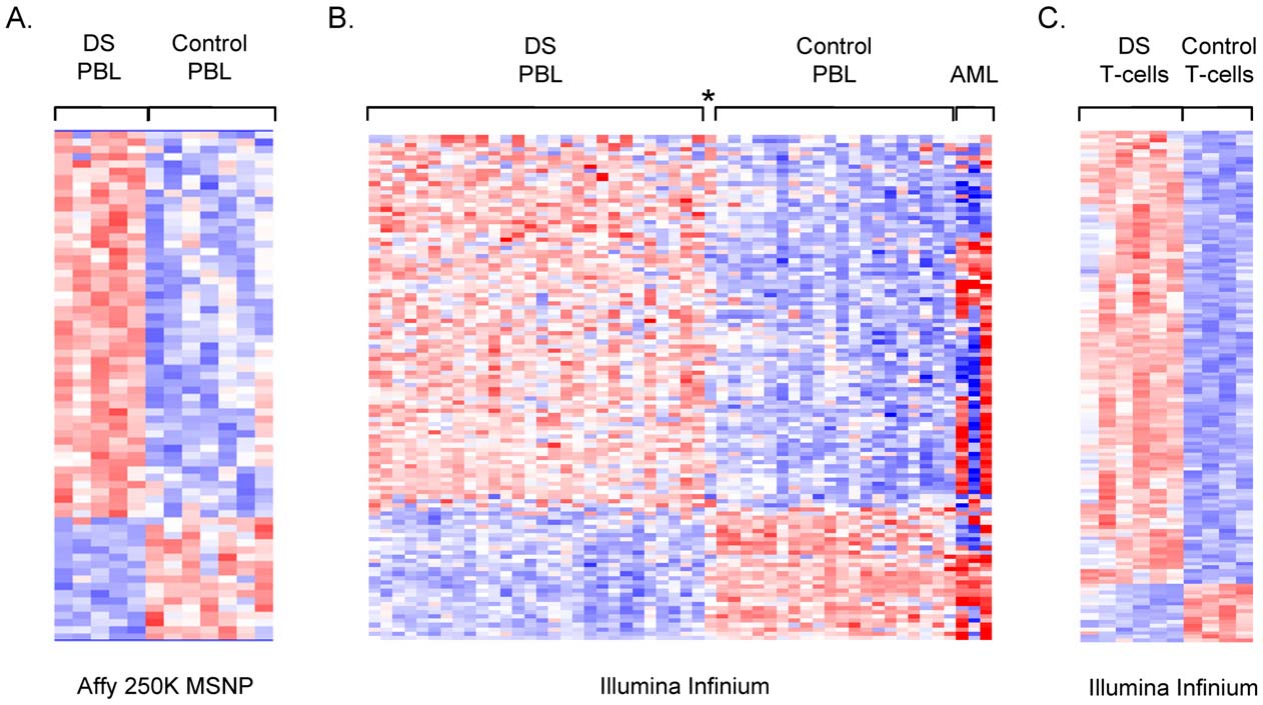
Microarray analysis of DNA methylation in DS versus normal PBL. A, Supervised hierarchical clustering of the MI values from Affymetrix 250 K *Sty*I MSNP. Applying ANOVA (p<.01) and a fold-change criterion (>1.2 fold change in MI in DS versus normal PBL) produced a set of 70 differentially methylated loci. The MI values for these loci were subjected to hierarchical clustering in dChip. Biological samples are on the x-axis and SNPs are on the y-axis with strong methylation indicated by the red color and weak or absent methylation by the blue color. B, Supervised hierarchical clustering of the Illumina Infinium data (fractional methylation) for 108 genes (118 probes) that passed ANOVA at p<.01, with additional criteria of >1.2 fold change and >0.1 absolute difference in DS versus normal PBL. The AML cases were not included in the statistical analyses but are shown here to highlight the fact that the perturbations in methylation in DS versus normal PBL are different and smaller in magnitude (less intense red and blue color) than in normal PBL versus AML. The asterisk indicates the single case of mosaic DS, which shows a pattern of methylation intermediate between DS and controls. C, Supervised hierarchical clustering of the Illumina Infinium data (percent methylation) for 140 CpGs, located in 134 different genes that passed ANOVA at p<.01 and the fold-change criterion (>1.3 fold change and >.15 absolute difference) in fractional methylation in DS T-cells versus normal T-cells. Genes mapping to the X or Y chromosomes were removed from each dataset (see Materials and Methods).

To begin to ask whether PBL from adults with DS might differ epigenetically from this same tissue in normal adults we first profiled DS and normal control samples for DNA methylation genome-wide on high density microarrays, using 2 complementary platforms: MSNP and Infinium BeadChip assays. The MSNP method adapts Affymetrix SNP arrays for methylation analysis by incorporating an initial methylation-sensitive restriction digestion [16], and queries the methylation status of CpG dinucleotides in *Hpa*II restriction sites roughly equally spaced along all human chromosomes in intragenic and intergenic regions (26,800 SNP-tagged loci reliably informative for CpG methylation in this experiment; see Methods). In contrast, the Infinium methylation assay utilizes bisulfite conversion of the genomic DNA and queries the percent methylation at each of 27,000 CpG dinucleotides concentrated in promoter regions of 14,000 human genes. We used MSNP on 250 K *Sty*I SNP arrays to compare 5 DS PBL samples (4 individuals; one sampled at 2 time points 6 years apart) to 7 normal control PBL (7 individuals). After calculating the methylation indices (MIs; see Materials and Methods) and carrying out non-supervised hierarchical clustering of these data we could not distinguish the DS from the control PBL samples, suggesting that trisomy 21 does not cause widespread changes in DNA methylation in PBL. However, when we analyzed the methylation values by ANOVA followed by supervised hierarchical clustering, we found small sets of candidate loci with consistent differences in methylation in DS versus normal PBL (Figure 1 and Table S1).

We applied Infinium BeadChip methylation assays to a larger set of PBL DNA samples, comparing 29 individuals with DS to 20 normal controls spanning the same age range. This larger dataset included all the samples that we had run by MSNP plus additional cases and controls. Similar to the findings with MSNP, non-supervised hierarchical clustering of the methylation values (percent methylation at each CpG queried by the array) showed no evidence for widespread alterations in DNA methylation in DS (not shown), but again ANOVA and supervised clustering produced a list of candidate differentially methylated loci (Figure 1 and Table S2). The differentially methylated loci in DS versus normal PBL from both of these microarray screens were found to be distributed on most of the human autosomes, with no specific enrichment for genes on chromosome 21 (Tables S1 and S2).

The reliability of the Illumina Infinium data was shown by extremely close correlations in technical replicates (examples of x-y plots and correlation coefficients in Figure S1). To further test reliability, and to ask whether the sets of differentially methylated loci clearly distinguish between DS and normal PBL we classified samples using a logistic ridge regression and validated the robustness of the classification scheme using leave-one-out cross-validation. Ridge regression was chosen for the ability of this approach to control for the colinearity of the independent variables. Leave-one-out cross-validation demonstrated that the differentially methylated loci from the Infinium screen classified DS versus normal PBL with 100% sensitivity and specificity. Strikingly, we initially observed one apparently mis-classified sample among the 29 cases of DS (asterisk in Figure 1), but this individual proved to have mosaic trisomy 21, with less than 50% trisomic (+21) cells by karyotype. None of the other individuals analyzed in the microarray experiments showed mosaicism.

This statistical approach was not suitable for the MSNP screen with the smaller set of samples, but we directly tested and validated the results for 3 differentially methylated loci from MSNP, as well as 7 loci from the Infinium screen, using bisulfite sequencing and/or MS-Pyroseq (see below). As a further technical consideration, single nucleotide polymorphisms (SNPs) in the regions probed by the Infinium assays can in principle complicate the results for a minor subset of loci. However, the absence of common annotated SNPs detected in the candidate regions from our screen (dbSNP; http://www.ncbi.nlm.nih.gov/), the representation of several of the differentially methylated loci by multiple probes on the BeadChips and, more directly, our successful direct independent validations by bisulfite sequencing and MS-Pyroseq (see below) indicated that most if not all of these loci are true positives.

### Validations of the microarray data by bisulfite sequencing

**Figure 2:**
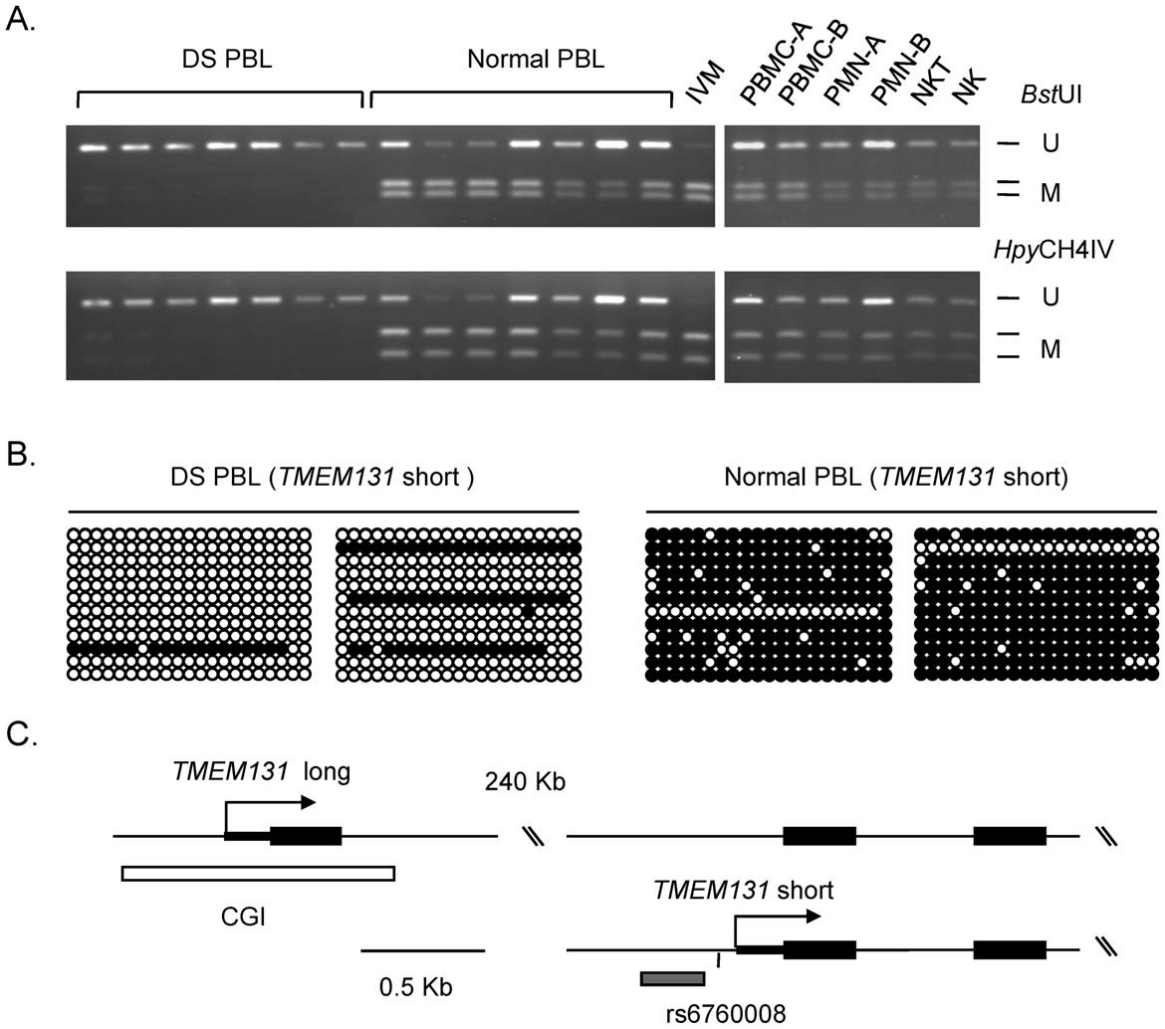
Validation of differential DNA methylation in the *TMEM131* gene in DS versus normal PBL. A, COBRA showing markedly less methylation in DS compared to normal PBL (left panel). The right panel shows COBRA results for PBMC, PMN, NK-T and NK cell fractions from normal blood. None of these lanes show the pattern seen in DS PBL, indicating that the loss of methylation in DS PBL is not due to changes in the proportions of these normal cell populations. U, unmethylated; M, methylated. IVM, in vitro methylated DNA. B, Bisulfite sequencing showing that the hypomethylation in the promoter of the short isoform of *TMEM131* in DS affects a large number of contiguous CpGs. C, map of the *TMEM131* locus, showing the major alternative transcripts. The differential methylation is in the promoter of the short isoform (grey bar, near the index SNP rs6760008); bisulfite sequencing of the far upstream CGI showed absence of methylation in both DS and controls (white bar; data in Figure S6).

Next we sought to validate the gene-specific differential methylation in the same DS cases and normal controls using the independent and definitive methods of combined bisulfite restriction analysis (COBRA) and bisulfite sequencing. These validations were successful for 10/10 loci chosen from the lists of differentially methylated genes that had passed our ANOVA and fold-change criteria in the microarray data (3 loci from the MSNP screen and 7 loci from the Infinium screen). Importantly, the bisulfite sequencing showed that for each gene the differential methylation affected not only the index CpG sites queried on the microarrays but also multiple adjacent CpG dinucleotides (examples in Figure 2, Figures S2, S3 and S4).

### Validations of differential methylation in DS versus control PBL by MS-Pyroseq

**Figure 3:**
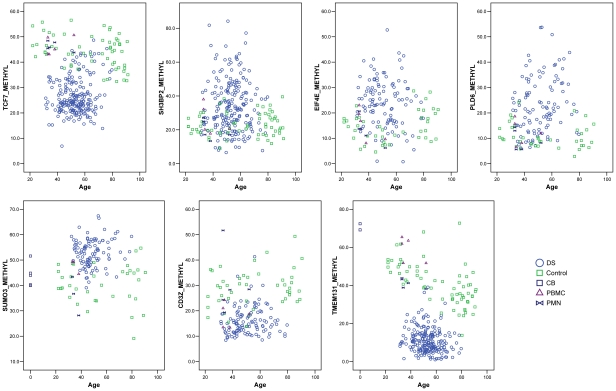
Quantitative MS-Pyroseq showing recurrent hypomethylation in the *TMEM131* internal promoter region, *TCF7* upstream region, and *CD3Z* promoter region, and hypermethylation in the *EIF4E*, *SH3BP2*, *SUMO3*, and *PLD6* promoter regions, in DS compared to normal PBL. Genomic DNAs were bisulfite converted and subjected to PCR followed by MS-Pyroseq. The values for percent methylation are averaged over multiple CpG dinucleotides in each region, as listed in Table S7. Mean values for methylation and the p-values from logistic regression adjusting for age are in Table 1. DS, total PBL from adults with DS; Control, total PBL from normal adults; CB, total leukocytes from normal umbilical cord blood; PBMC, normal adult peripheral blood mononuclear cells; PMN, normal adult peripheral blood polymorphonuclear leukocytes.

We next sought to determine the frequency and specificity of differential methylation in a larger series of individuals. To this end we used MS-Pyroseq, which measures the percent methylation at multiple CpGs downstream of the sequencing primer. We applied this assay to 9 of the candidate loci. For 8 of these loci, *TMEM131*, *TCF7*, *SH3BP2*, *SUMO3*, *CPT1B*, *CD3Z*, *EIF4E* and *PLD6* (LOC201164) we found strikingly different distributions of methylation values (percent methylation averaged over multiple contiguous CpGs) in DS compared to normal PBL (Table 1 and Figure 3 and Figure S2). The distributions of methylation values were largely dichotomous, with only minimal overlap between DS and controls, for *TMEM131*, *PLD6*, *EIF4E*, *CPT1B* and *CD3Z*, while for *TCF7*, *SUMO3*, and *SH3BP2* the distributions were more overlapping but nonetheless showed a clear shift in the mean values in DS versus controls. For all of these genes the inter-group differences in methylation were highly statistically significant (p<.0001; Table 1). MS-Pyroseq for the ninth gene tested, *FAM62C*, revealed a wide range of methylation in both groups (DS and controls) but the distribution of methylation still differed significantly, albeit less strongly, between the 2 groups (Table 1). In summary MS-Pyroseq in the larger case-control series validated the differential methylation for each of these 9 candidate loci, 3 from MSNP and 6 from the Infinium assays, thus giving high confidence in the quality of the primary methylation profiling data.

**Table 1:** Genes validated by MS-Pyroseq as differentially methylated in DS versus normal PBL.

Gene Name	Affy SNP ID or Illumina CG ID	Mean % methylation; including multiple time points	Mean % methylation; single time points only	Gene Product and Function	Tissue-Specific Expression^a^
*TMEM131*(Chr2)	SNP_A-2136365; Internal promoter region (CG-rich but not meeting criteria for CGI^b^)	DS, 10.9(n = 207)Control, 41.1(n = 73)p<.0001	DS, 10.9(n = 186)Control, 40.8(n = 67)p<.0001	Transmembrane protein (long isoform); structure of smaller protein isoform(s) unknown.	Highest expression in spleen and thymus. Strongly expressed in hematopoietic stem cells, myeloid precursor cells, NK, T- and B-lymphocytes. Expression in NK cells induced by IL-2/PHA. Expression increases between the multipotent progenitor and pro-T cell stage.
*TCF7*(Chr5)	SNP_A-2179466; conserved CG-rich region 5 kb US of promoter CGI	DS, 26.5(n = 207)Control, 44.0(n = 64)p<.0001	DS, 26.6(n = 191)Control, 44.2(n = 59)p<.0001	Transcription factor (a.k.a. TCF-1); role in Wnt signaling pathway; essential for NK cell development.	Highest expression in whole thymus, NK cells and T cells. Expression in NK cells is suppressed by IL-2/PHA. Expression increases strongly between the multipotent progenitors and pro-T cell stage.
*SH3BP2*(Chr4)	cg07991621 and cg08822227; promoter CGI	DS, 34.5(n = 203)Control, 23.6(n = 67)p<.0001	DS, 34.9(n = 185)Control, 23.0(n = 64)p<.0001	Cytoplasmic protein; signaling adaptor. Essential for normal B-cell and NK cell function.	Highest expression in blood cells, including B cells, T cells, NK cells, monocytes and myeloid precursor cells. Expression in NK cells induced by IL-2/PHA.
*FAM62C*(Chr3)	SNP_A-1974529; 0.5 kb US of promoter CGI	DS, 46.0(n = 87)Control, 40.1(n = 45)p = .005	DS, 46.1(n = 81)Control, 39.8(n = 40)p = .005	C2-domain protein; synaptotagmin family. Precise function not known.	Highest expression in lung and brain; weaker expression in hematopoietic cells.
*CD3Z*(*CD247*)(Chr1)	cg09554443;first exon; no CGI	DS, 16(n = 113)Control, 29(n = 45)p<.0001	DS, 16.3(n = 90)Control, 29(n = 45)p<.0001	T-cell receptor zeta chain isoform 2 precursor. Crucial for T-cell signaling.	High expression in whole blood, NK cells, T cells, B cells, thymus and bone marrow.
*PLD6*(LOC201164)(Chr17)	cg05590257;promoter CGI	DS, 23.1(n = 105)Control, 10.8(n = 40)p<.0001	DS, 23.4(n = 88)Control, 10.9(n = 39)p<.0001	Phospholipase D6. Precise function not known.	Widely expressed.
*EIF4E* (Chr4)	cg14972143; 500 bp US of promoter CGI	DS, 24(n = 110)Control, 14.7 (n = 42)p<.0001	DS, 23.7(n = 87)Control, 14.7 (n = 42)p<.0001	Eukaryotic translation initiation factor 4E. Essential for efficient translation of cell proliferation-related genes.	Highest expression in bone marrow CD34+ hematopoietic stem cells and in neural tissues.
*CPT1B*(Chr22)	cg00983520; promoter CGI	DS, 71.1(n = 73)Control, 46.2(n = 33)P<.0001	DS, 71.9(n = 58)Control, 46.2(n = 33)P<.0001	Carnitine palmitoyltransferase 1B. Metabolic enzyme.	Highest in heart, testis, CD34+ hematopoietic cells, T cells and B cells.
*SUMO3*(Chr21)	cg21053323; promoter CGI	DS, 53(n = 98)Control, 40.7 (n = 39)p<.0001	DS, 53.3(n = 75)Control, 40.3 (n = 36)p<.0001	Small ubiquitin-like modifier protein 3. Multiple functions exerted via post-translational protein modifications.	High expression in bone marrow CD34+ hematopoietic cells, NK cells, B cells, dendritic cells and monocytes.

The p-values are for differential methylation from logistic regression adjusting for age. These calculations are shown both for the series including individuals with DS who provided PBL samples at multiple time points from 6 months to 7 years apart, and for same series of individuals narrowed to include only the first blood draw. As shown here, all 9 candidate loci tested by MS-Pyroseq showed highly significant differential methylation concordant with the microarray data. These results give strong confidence in the overall accuracy of the microarray data; complete lists of differentially methylated loci are in Tables S1 and S2.

a Expression summaries are from the GNF Expression Atlas 2 microarray data (http://expression.gnf.org/cgi-bin/index.cgi), Unigene (http://www.ncbi.nlm.nih.gov/UniGene/ESTProfileViewer.cgi), and microarray data in NCBI/GEO accession GDS751, see [25]. HSC, CD34+ hematopoietic stem cells.

b CGI criteria are: GC content of 50% or greater; length greater than 200 bp; ratio greater than 0.6 of observed number of CG dinucleotides to the expected number on the basis of the number of Gs and Cs in the segment.

### Age-dependence and stability of the gene-specific differential methylation

As methylation is known to be age-dependent for certain DNA sequences in some human tissues including the immune system [17], for 8 of the independently validated differentially methylated genes we plotted the percent methylation in DS cases and controls as a function of age (Figure 3 and Figure S2). For *TMEM131*, the average percent methylation clearly declined with age in the normal controls, but was uniformly low regardless of age in the adults with DS (Figure 3). Despite the decline with age in the controls, and our deliberate sampling of PBL from both young and elderly control individuals, the levels of methylation in this region, corresponding to a CG-rich internal promoter sequence of the *TMEM131* gene, never reached the very low levels seen in DS. Similar analyses showed that for all 8 candidate genes tested the difference in methylation between DS and controls was significant both before and after adjusting for age (Table 1). As we had collected PBL samples from several of the study participants with DS at multiple time points spanning from 6 months up to 7 years, we were further able to ask whether the methylation abnormalities were stable over time in these individuals. As shown in Table S3, the degree of methylation as determined by MS-Pyroseq was generally stable over time.

Mosaicism for trisomy 21 can be found in a minor subset of individuals with DS, and this finding has been associated with less severe phenotypes. The large majority of DS cases in our series had complete trisomy 21, but 3 cases (one of which was run on the Infinium BeadChips and all three of which were analyzed by MS-Pyroseq) showed moderate to high mosaicism with cells disomic for chromosome 21 constituting >15% of the leukocytes in the peripheral blood. While the rarity of these mosaic cases precluded a statistical analysis, as shown in Table S4 the cases with the greatest percentage of normal diploid cells (high level mosaics) showed methylation values closer to the normal range.

### The abnormal DNA methylation in DS PBL is not due to altered cell counts

Abnormalities of B- and T-lineage lymphocytes, either functional, numerical or both, have been reported in children and adults with DS [18]–[25]. We therefore considered whether grossly altered blood cell differential counts with normal cell type-specific variation in DNA methylation might trivially account for our findings of altered methylation in DS. We first performed automated complete blood counts for 4 of the DS blood samples, all of which showed strongly reduced methylation of the internal promoter of *TMEM131* and the upstream region of *TCF7* and increased methylation of the upstream portion of the *SH3BP2* CGI. We found that the numbers and percentages of polymorphonuclear leukocytes (PMNs) and total lymphocytes were all within the normal range for our clinical laboratory (Table S5). The percentages of monocytes were slightly increased (range in the 4 DS cases 6.4–11.9 percent; normal range 4–8 percent, Table S5) but as measurements of DNA methylation linearly average over all cells in a given sample, this slight increase in a minor cell population would not be sufficient to account for the altered methylation in DS PBL. We next fractionated several normal PBL samples into mononuclear cells (PBMCs) versus PMNs on Ficoll gradients and performed COBRA and MS-Pyroseq on the genomic DNAs. This analysis revealed cell type-specific methylation levels, but we found no evidence for differences in methylation of *TMEM131*, *SH3BP2*, *EIF4E*, or *TCF7* between these 2 cell populations with a direction and magnitude that could account for the altered methylation observed in DS, even if cell numbers were altered (Figure 2A and Figure S4). Only for one of the differentially methylated genes, *CD3Z*, did we observe a difference in methylation in PBMC compared to PMN with a magnitude and direction that could possibly account for the observed differential methylation in DS versus normal PBL based on abnormal lymphocyte numbers. However, we were able to exclude this trivial explanation for *CD3Z* by showing that its CpG methylation is specifically altered in purified T-cells from DS versus controls (below). Lastly, given that one of the reported findings in adults with DS is an increase in the minor sub-population of T-lineage lymphocytes with the immunophenotype of natural killer (NK) cells, we did a further control assessing the methylation of *TMEM131* in DNA from NK cells immunopurified from normal individuals. This analysis showed that the critical region of the *TMEM131* gene is in fact slightly *hyper*methylated in normal NK cells; a pattern opposite to the *hypo*methylation seen in whole blood from DS (Figure 2A). Similar results excluding the possibility that the observed alterations in methylation might be trivially due to increased numbers of normal NK cells were obtained for the *TCF7* and *SH3BP2* genes (Figure S4).

### Gene-specific abnormalities in DNA methylation in T-lymphocytes from DS

To directly assess CpG methylation within an important lymphocyte subset, we next prepared genomic DNA from T-cells immuno-purified from PBL of 12 individuals with DS and 15 control individuals, and measured *SUMO3*, *CD3Z* and *SH3BP2* promoter methylation, as well as methylation of the *TMEM131* internal promoter region, by MS-Pyroseq. This procedure confirmed that all of these loci are differentially methylated between DS and normal controls, not only in whole PBL but also in the T-cell preparations, thus arguing against our findings in DS PBL being trivially due to altered numbers of (epigenetically normal) T-cells (Figure S5). To obtain genome wide methylation data in this cell type, we next profiled promoter methylation in T-cell DNAs from 4 normal adults and 6 adults with DS for which sufficient DNA was available, using the Infinium BeadChips. Similar to our findings using total PBL, ANOVA followed by supervised clustering of the methylation values revealed a small set of differentially methylated loci (140 CpGs, located in 134 different genes) in this DS versus normal T-cell comparison (Figure 1 and Table S6). Among the 108 genes (118 probes) identified as differentially methylated in our Infinium data from DS versus normal total PBL, a large subset, 17 genes (19 probes), were also found to be differentially methylated in this genome-wide analysis with the T-cell preparations. This observation of gene-specific differential methylation in purified T-cells from DS versus control individuals further supports our conclusion that the epigenetic changes reported here reflect bona fide abnormalities within specific cell types and cannot be trivially accounted for by altered percentages of the major types of leukocytes.

### Altered expression of *TMEM131*, *TCF7*, and *NPDC1* in DS PBL

**Figure 4:**
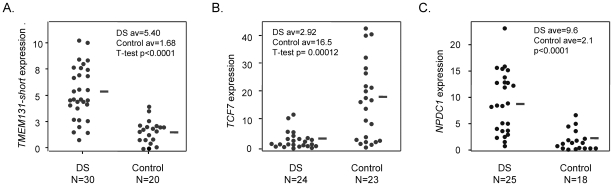
Altered mRNA expression of *TMEM131*, *TCF7*, and *NPDC1* in DS versus normal PBL. A, Expression of *TMEM131* short isoform mRNA measured by Q-PCR. The difference between DS and normal controls is highly significant (T-test, p<0.0001). B, Expression of *TCF7* mRNA measured by Q-PCR. The difference between DS and normal controls is highly significant (T-test, p = .00012). C, Expression of *NPDC1* mRNA measured by Q-PCR. The difference between DS and normal controls is highly significant (T-test, p<0.0001). The Q-PCR primers are listed in Table S7. The *TMEM131* Q-PCR primers are specific for the short mRNA isoform; the *TCF7* Q-PCR primers amplify both major isoforms of *TCF7* mRNA. The *NPDC1* Q-PCR primers amplify all major isoforms.

DNA methylation in *cis*-acting regulatory sequences can affect gene transcription, with hypermethylation of CG-rich promoter regions causing or consolidating transcriptional repression and methylation in insulator or repressor elements sometimes causing an opposite phenomenon of increased gene expression. Total RNA was available from some, though not all, of the PBL samples in this study, and we measured mRNA transcript levels in these samples by Q-PCR. As shown in Figure 4, isoform-specific Q-PCR revealed, as predicted, greater amounts of mRNA initiating from the internal promoter region of *TMEM131* in the DS PBL samples (in which this region is relatively *hypo*methylated) compared to normal PBL (in which this region has substantial methylation). In contrast, expression of the longer *TMEM131* mRNA isoform initiating from the upstream CGI-associated promoter, which was uniformly unmethylated in both DS and normal PBL, did not differ between these 2 groups (Figure S6). As a second example, *NPDC1* was among the genes that showed consistent promoter *hypo*methylation in DS compared to normal PBL by the Infinium assays (Table S2) and Q-PCR for *NPDC1* mRNA revealed that the DS PBL samples showed, on average, greater expression of this gene than the normal PBL samples (Figure 4). A third example was *TCF7*, for which Q-PCR revealed that DS PBL samples have, on average, significantly less expression of mRNA from this gene than the normal PBL samples (Figure 4). This somewhat unexpected finding suggested that the evolutionarily conserved region 5 kb upstream of the *TCF7* promoter, which is *hypo*methylated in many of the DS PBL samples (Table 1, Figure 3 and Figure S2), might be acting as a negative regulatory element with greater repressive function when hypomethylated. Further insight was obtained by assessing DNA methylation directly at the upstream border of the promoter-associated CGI of this gene by MS-Pyroseq, which revealed a statistically significant tendency toward *hyper*methylation in DS compared to normal PBL (Figure S7). Thus individuals with DS often have substantial *hypo*methylation of the conserved region 5 kb upstream of the *TCF7* transcriptional initiation site, and also show a significant though weaker trend toward *hyper*methylation of the upstream border of the *TCF7* CGI, located closer to the transcription initiation site.

Overall, these data are consistent with regulation of mRNA expression at these 3 loci by DNA methylation – a conclusion further supported by functional experiments using a demethylating drug (below). However, in interpreting the relatively wide range of expression in the primary PBL samples it is important to take into account that the *TMEM131*, *TCF7* and *NPDC1* genes are all known to be highly inducible in response to signaling in NK cells and probably in other lymphocyte classes (microarray data in NCBI/GEO, accessions GDS751 and ref. [26]), so the net expression in a given sample is likely determined by the interaction between acute environmental signals (cytokines and cell-cell interactions) and the baseline methylation status of the locus. Consistent with the acute inducibility of these genes and hence the wide range of expression in primary blood samples from both DS and normal individuals, the differences in expression that we observed were highly statistically significant in the overall comparisons between DS and normal controls (Figure 4), and hence correlated strongly on average with the extent of methylation, but we could not detect strong correlations between the extent of methylation and the expression level among individuals within each group.

### Recapitulation of the abnormal gene expression by exposing normal human T-cells to the demethylating drug 5aza-dC

**Figure 5:**
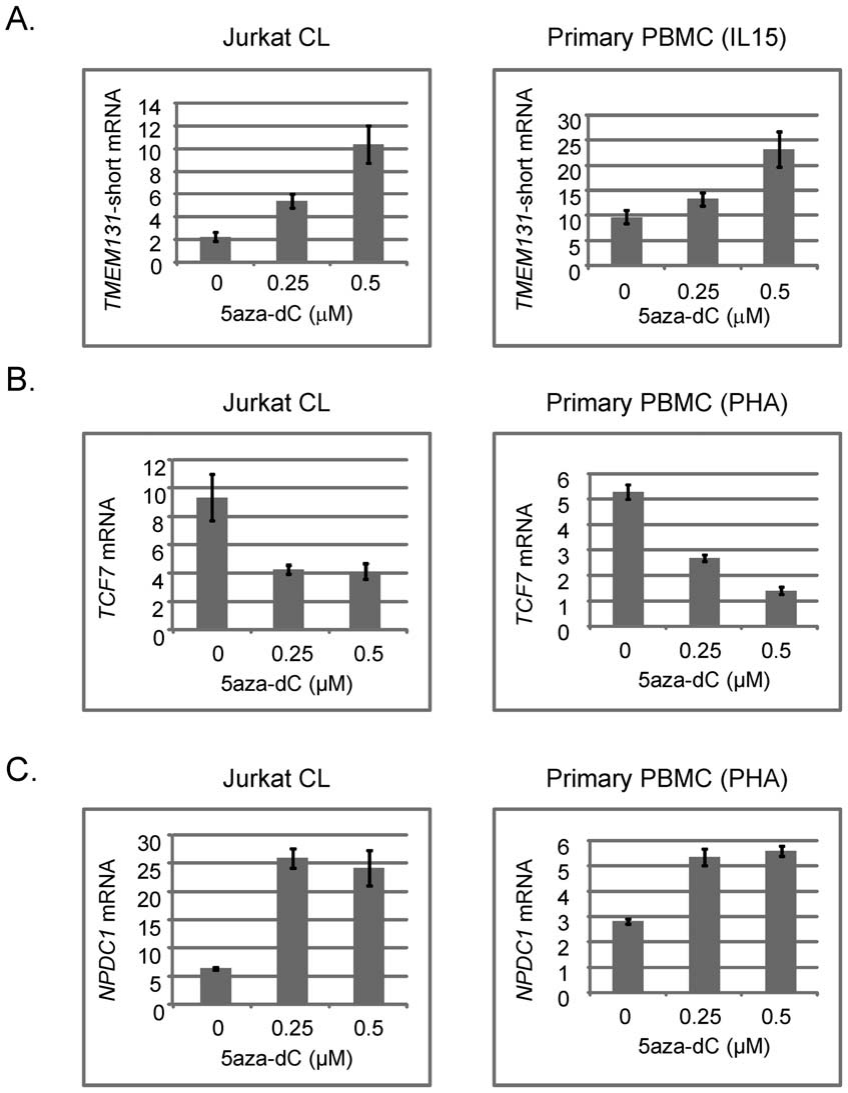
Effects of the demethylating drug 5aza-dC on expression of *TMEM131* short isoform, *TCF7*, and *NPDC1* mRNAs in Jurkat cells and normal human T-cells expanded with mitogens. A, Increased expression of *TMEM131* short isoform mRNA in Jurkat cells and PBMC exposed to 5aza-dC at the indicated concentrations for 3 days. The short-term cultures of PBMC were grown in the presence of IL-15 to induce proliferation of cytotoxic T-cells and NK cells. B, Decreased expression of *TCF7* mRNA in Jurkat cells and PBMC exposed to 5aza-dC at the indicated concentrations for 3 days. The short term cultures of PBMC were grown in the presence of PHA to induce a polyclonal proliferation of T-cells. PHA rather than IL-15 stimulation was utilized for assessing *TCF7* expression and response to 5aza-dC because the baseline expression of *TCF7* mRNA is high after PHA stimulation but very low after stimulation with IL-15. C, Increased expression of *NPDC1* mRNA in Jurkat cells and PBMC exposed to 5aza-dC at the indicated concentrations for 3 days. The short term cultures of PBMC were grown in the presence of PHA to induce a polyclonal proliferation of T-cells. In each experiment a 25 – 40 percent reduction in DNA methylation of the index regions of interest after exposure to the highest dose of 5aza-dC was confirmed by MS-Pyroseq or bisulfite sequencing (data not shown). Cell viability was preserved in each experiment, but net cell proliferation was reduced by approximately 50% at the highest doses of 5aza-dC.

**Figure 6:**
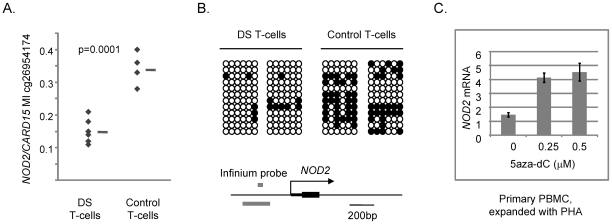
Differential DNA methylation in the *CARD15/NOD2* gene in DS versus normal T-cells and induction of *NOD2* mRNA by 5aza-dC. A, Fractional methylation values from the Infinium assays showing differential methylation at cg26954174 in the *NOD2* promoter region in DS versus control T-cells. B, Bisulfite sequencing showing relative hypomethylation in the (non-CGI) promoter region of *NOD2* in DS T-cells. The map of the *NOD2* promoter region shows the location of the Infinium probe (grey bar above the line, cg26954174); the region subjected to bisulfite sequencing is shown by the grey bar below the line. C, Increased expression of *NOD2* mRNA in PBMCs exposed to 5aza-dC at the indicated concentrations for 3 days. The short term cultures of PBMCs were grown in the presence of PHA to induce a polyclonal proliferation of T-cells.

To test more directly for a functional relationship of methylation with alterations in gene expression we examined *TMEM131*, *TCF7*, *NOD2*, *SUMO3*, *CPT1B*, *CD3Z* and *NPDC1* mRNA expression in a well controlled cell culture system using the demethylating drug 5aza-dC. We exposed a proliferating T-cell line (Jurkat) and, more importantly, normal PBMCs isolated from fresh peripheral blood samples and expanded with a cytokine (IL-15) that induces proliferation of cytotoxic T-lymphocytes and NK cells, or with a general T-cell mitogen (phytohemaglutinin; PHA), to 5aza-dC for 3 days. We then prepared DNA and RNA from these cells and measured DNA methylation and mRNA expression of these 7 genes. In the absence of drug we found more robust expression of *TMEM131* when the PBMCs were expanded with IL-15, while expression of *TCF7*, *NOD2* and *NPDC1* was higher when these cells were expanded with PHA. All 4 genes were readily detected in the proliferating Jurkat cells without cytokines. In experiments using the appropriate mitogens (IL-15 for analyzing *TMEM131* and PHA for analyzing *TCF7*, *NOD2* and *NPDC1*) we found that *TMEM131* short isoform mRNA, *NOD2* mRNA and *NPDC1* mRNA levels increased, while *TCF7* mRNA levels decreased, as a function of exposure to 5aza-dC, both in the Jurkat cells and in the primary PBMCs (Figure 5 and Figure 6). Also shown in Figure 6 are our independent validations of the Infinium data for *NOD2* by bisulfite sequencing, which confirm the relative loss of methylation in T-cells from DS patients, compared to T-cells of normal adults in the (non-CGI) promoter region of this gene. These data are consistent with a functional role for DNA methylation in modulating the expression of these genes in lymphocytes (and possibly in monocytes as well) and for the 3 genes from the PBL screen the directions of their changes in expression upon demethylation (increased for *TMEM131* and *NPDC1* and decreased for *TCF7*) match the predictions based on the differences of their average expression levels in DS versus normal PBL (Figure 4). For another two genes, *NOD2* and *CD3Z*, our analysis of mRNA expression is still in progress but the aggregate results so far are consistent with these two genes being functionally regulated by promoter methylation. The DNA methylation data (both genes hypomethylated in DS T-cells compared to normal T-cells) and 5aza-dC response (significantly increased expression of both genes on exposure of normal PHA-stimulated PBMC to the demethylating drug) are consistent with a functional role for CpG methylation in down-modulating expression of these genes in normal T-cells (Figure 6 and data not shown). Furthermore, in a small number of purified T-cell samples analyzed so far (7 DS T-cell and 8 normal T-cell preparations) both of these genes are over-expressed on average in the DS T-cells compared to normal T-cells (2-fold for *NOD2* and 2.5-fold for *CD3Z*). However, these Q-PCR data have shown high inter-sample variability and have not yet reached statistical significance by T-tests.

In contrast to the positive data obtained for *TMEM131*, *TCF7*, *NOD2*, *CD3Z* and *NPDC1*, the two remaining differentially methylated genes that we tested, *SUMO3* and *CPT1B*, have shown negative data or paradoxical correlations between CpG methylation, mRNA expression and response to 5aza-dC. For the *SUMO3* gene, located on chromosome 21, we found by Q-PCR that the mean expression level in DS PBL is about 1.5-fold greater than in normal PBL, thus being consistent with a simple physical gene dosage effect, with no obvious compensatory effect of the promoter hypermethylation (data not shown). For the *CPT1B* gene Q-PCR revealed a paradoxical correlation between promoter hypermethylation and significant over-expression of *CPT1B* mRNA in DS PBL. This paradoxical relationship was not clarified by examining the response of *CPT1B* transcription to 5aza-dC, as the demethylating drug caused a slight increase, not a decrease, in its mRNA levels both in Jurkat cells and in normal PBMC (data not shown).

## Discussion

The primary cause of DS, namely trisomy 21, has been known since 1959, but the pathogenesis of the diverse phenotypic features of this syndrome, not only in brain and cardiac development but also in a range of blood cell-related phenotypes including macrocytic anemia, autoimmunity, and recurrent infections, remains incompletely understood. Profiling of mRNA in cells and tissues with +21 has revealed widespread changes in gene expression, mostly small in magnitude, both for genes on chromosome 21 and for large groups of genes on other chromosomes. However sorting out the importance of any given gene has been difficult. Mice with partial trisomies, transgenic mice, and recently mice engineered to carry human chromosome 21, are useful experimental tools for assigning or excluding roles of specific genes and regions on chromosome 21 in conferring the diverse features of DS [27]. But additional experimental approaches are needed to understand the complex genomic, cellular and tissue response to this simple chromosomal aneuploidy.

Previously Chango et al. used a combination of methylation-sensitive arbitrarily primed polymerase chain reaction (MS-AP-PCR) and quantitation of DNA fragments to find 6 fragments that were hypermethylated in PBL from 8 individuals with DS, compared to 8 normal controls [28]. The authors suggested that the observed differences might provide a mechanism to silence constitutively over expressed genes in DS, but the methods did not allow the DNA sequence of these fragments to be determined. Here we have taken a genome-wide screening approach using 2 independent platforms that are entirely distinct in how they query methylation and are complementary and largely non-overlapping in their coverage of CpG sites. MSNP uses methylation-sensitive restriction digestion as the initial step to query the methylation status of CpG dinucleotides in *Hpa*II restriction sites in intragenic and intergenic regions, most of which are not promoter-associated; in contrast the Infinium methylation assay utilizes bisulfite conversion of the genomic DNA as the initial step and queries the percent methylation of CpG's in promoter regions, including many CpG islands as well as a large number of non-island promoter sequences. These genome-wide microarray-based screens, with validations by independent methods in a larger series of DS cases and controls, show that there are highly recurrent gene-specific epigenetic changes in this common chromosomal disorder.

Our results are from analyzing blood cells, so it is important to consider what is known about the effects of trisomy 21 in this cell lineage. There are indications from studying mosaicism over time that trisomy 21 is weakly but continually selected against in hematopoietic cells [29]–[31]. Related findings in well controlled mouse models include strongly reduced growth capacity of bone marrow stem cells in the partial trisomy Ts65Dn model of DS [32], defects in hematopoietic progenitor cells and macrocytosis in a related partial trisomy mouse model, Ts1Cje [33], as well as hematopoietic abnormalities in the more recently created Tc1 trans-chromosomal model [34]. Combining these observations with our current data, one possibility is that there may be biological selection over time for specific patterns of altered DNA methylation in hematopoietic stem/progenitor cells that affect net cellular proliferation in this aneuploid genetic background. Ongoing biological selection acting on stochastic variations in DNA methylation could result in altered DNA methylation, as observed here, and changes in biological properties, such as the reported functional abnormalities in NK cells [22]. In considering alternative explanations for our findings, major changes in the DNA methylation machinery are less likely, given that the microarray data show only gene-specific and not widespread alterations in DNA methylation.

Immunological abnormalities are prominent in DS, and many of the differentially methylated genes in Table 1, Tables S1 and S2 have known or predicted roles in the immune system. Among the genes that we have focused on for downstream analyses in this study several (*TCF7*, *SH3BP2*, *CD3Z* and *NOD2*) are already known to be essential for normal lymphocyte development and function, while another group (*TMEM131*, *PLD6*, *NPDC1*and *EIF4E*) are interesting candidates for such a role. *TMEM131* methylation in the internal promoter region is strikingly and consistently different in DS versus normal PBL, and this gene, encoding a transmembrane protein, has been shown to be cytokine-inducible in NK cells, together with another gene that we have studied here, *NPDC1* (microarray data in NCBI/GEO, accession GDS751; ref. [26]). These genes are therefore intriguing biological candidates for contributing to the pathogenesis of NK cell defects in DS. Little information is available on the function of *TMEM131* but this gene was reported to be sharply up-regulated between the multipotent progenitor and pro-T cell stages of differentiation, along with only a few other genes, strikingly including another gene which we have shown here to be abnormally methylated in DS, namely the transcription factor gene *TCF7* [35], which is known to be essential for normal NK cell function [36]–[39]. While these genes are evidently co-regulated in development, they can respond oppositely to acute exposures to cytokines: expression of *TMEM131* and *NPDC1* in NK cells is up-regulated by IL-2/PHA while *TCF7* expression is down-regulated (microarray data in NCBI/GEO accession GDS751 [26]). This fact is interesting in view of the inverse abnormalities in expression of *TMEM131* and *NPDC1* (increased) compared to *TCF7* (decreased) in DS PBL, which is paralleled by the inverse responses of these genes to DNA demethylation in our experiments using 5aza-dC.

Considering the possible functions of some of the other differentially methylated genes, *NOD2* encodes a pathogen recognition receptor that is often studied in monocytes and macrophages but it is also expressed by CD34+ hematopoietic stem/progenitor cells and FOXP3-positive T regulatory cells [40]. *SH3BP2* codes for a pleckstrin homology domain- and Src homology 2 (SH2) domain-containing adaptor protein that is preferentially expressed in hematopoietic tissues including macrophages, NK, T-, and B-cells. It is involved in leukocyte signaling downstream Src/Syk-kinases and plays a crucial role in signaling during cell differentiation [41]. *PLD6*, encoding a member of the phospholipase-D family, has not yet been studied for its role in lymphocyte physiology but other phospholipase-D family members are known to be crucial for signaling downstream of the T-cell receptor [42]. *EIF4E*, encoding a translational initiation factor that is rate-limiting for expression of numerous proliferation-related cellular proteins, is up-regulated during T-cell activation and promotes lymphocyte chemotaxis [43], [44]. *SUMO3* is one of a family of small sumo proteins that modify the activities of other cellular proteins by post-translational sumoylation – a process that is known to affect lymphocyte physiology including regulation of immunoglobulin production by B-cells [45] and mitogenesis and cytokine production in T-cells [46], [47]. The biological role of the protein encoded by the *NPDC1* gene is not yet known, and knockout mice lacking this gene did not show an obvious developmental phenotype [48]. Based on our findings of altered methylation and expression of this gene in DS PBL, and the observation of its induction during NK cell activation noted above, it would be interesting to study immune system function in the *Npdc1*-deficient mice. Tables S1 and S2 contain additional examples of differentially methylated genes encoding cytokines, receptors and transcription factors that also warrant examination for roles in the normal immune system and in the immunological abnormalities associated with DS.

In summary, our findings show that recurrent gene-specific alterations in CpG methylation are a stereotypical cellular response to trisomy 21, with functional consequences in gene regulation. Interesting gene candidates for the immune dysfunction in DS are already emerging from these data, and as insights from studying DS as a model system have often shed light on physiological mechanisms in the general population it will also be important to dissect the roles in the normal immune system of the genes from our screen. Additional screens, including analysis of other tissues such as heart and brain, will be useful for pinpointing loci that are recurrently altered by gains or losses of DNA methylation in other cell types that contribute to key aspects of DS such as cardiac defects and cognitive disability. A more general corollary of our findings, beyond trisomy 21, is that there may be recurrent and predictable epigenetic consequences of other chromosomal copy number aberrations - for example in several types of human cancers, such as leukemias, Wilms tumors, and sarcomas, that frequently have simple aneuploid karyotypes.

## Materials and Methods

### Study subjects and diagnosis

This study was approved by Institutional Review Boards of the New York State Institute for Basic Research and Columbia University Medical Center. Participants with DS were ascertained through the New York State developmental disability service system as well as agencies in New Jersey, Connecticut and Northern Pennsylvania and have been assessed comprehensively including full medical chart reviews. The participants were recruited through responsible state and private service agencies, who contacted the participant's families or correspondents for permission for us to recruit. Informed consent was provided by either a parent or correspondent, and assent was obtained from the participant. The distribution of age, level of intellectual disability and residential placement did not differ between those participating and those who refused. Age-matched control participants were laboratory volunteers and participants in the Washington Heights-Inwood Community Aging Project who gave informed consent for genetic studies. Confirmation of trisomy 21 by G-banded karyotypes was available for 98% of the study participants with DS, with 100% concordance between cytogenetics and the clinical diagnosis of DS. Of those karyotyped, the large majority had complete trisomy 21. However, 7 cases exhibited low level mosaicism with most of the cells having trisomy 21 and less than 15% of the cells showing a diploid chromosome complement, 3 cases showed higher level mosaicism with greater than 15 percent of cells having 46 chromosomes (disomic for chromosome 21) and six cases presented with Robertsonian translocations, which in each case produced complete trisomy for the euchromatic region of chromosome 21 in all cells.

### MSNP and Infinium assays for profiling DNA methylation

MSNP on Affymetrix 250 K *Sty*I arrays was carried out essentially as previously described [16], [49], [50]. Each biological sample (total peripheral blood leukocyte DNA) was analyzed by hybridizing the arrays with genomic representations (probes) made according to the Affymetrix protocol, with the following pre-digestions of the genomic DNA as the first step in the procedure: *Sty*I (*S*), *Sty*I+*Hpa*II (*SH*), *Sty*I+*Msp*I (*SM*). All other steps subsequent to the genomic pre-digestions were according to the Affymetrix protocol. The *S*, and *SH* representations were prepared and hybridized in duplicate for each biological sample; the *SM* representations were single for each sample. Infinium Human Methylation27 BeadChip (Illumina) assays, based on bisulfite conversion of genomic DNA followed by primer extension on the BeadChips to query the methylation status of defined CpG dinucleotides, were performed according to the protocol from the manufacturer.

### Microarray data analysis

The MSNP data (.cel files) were processed in dChip ([51]; http://biosun1.harvard.edu/complab/dchip/) by normalization, model-based expression, and chromosome analysis. We assigned a numerical ploidy of 2 to the *S* arrays from the normal PBL samples, leaving the ploidy field blank for all other arrays. This strategy allowed us to visualize, using the chromosome view in dChip, the methylation status of *Hpa*II sites flanking a given SNP-tagged locus as the extent of reduction in signal intensity in the *SH* representations, compared to the *S* representations. As *Msp*I is the methylation-insensitive isoschizomer of *Hpa*II, the signal intensities observed in the *SM* representations allowed us to determine the reliability of the Class 2 SNPs (those with adjacent *Hpa*II sites thus informative for methylation status [16], [49], [50]), with reliable Affymetrix probe sets indicated by strong reduction in signal in *SM* compared to *S*. For the 26,800 Class 2 loci with *SM*_av_/*S*_av_<0.5 we calculated the methylation index (MI) as the fractional preservation of intensity in *SH* compared to *S*. Similar lists of candidate differentially methylated genes were obtained when we first subtracted *SM*_av_ as background and then calculated the methylation index.

The Infinium BeadChip data were processed using Genome Studio software, which calculates the percent methylation at each CpG queried by the arrays. The numerical values for methylation index (MSNP) and percent methylation (Infinium) were imported to dChip as external data and analyzed by ANOVA and supervised hierarchical clustering after removing all probes for genes on the X or Y chromosome, and applying fold-change and absolute difference criteria (Results and Figure 1 legend). To statistically validate the Infinium data we classified DS versus normal PBL using the % methylation of the differentially methylated loci in Table S2 using a logistic ridge regression. The ridge parameter was set to 10^−8^. Leave-one-out cross-validation was used to demonstrate that the classifier was not over trained to our particular test samples. The ridge regression, sensitivity, and specificity calculations were performed using Weka 3.4.

### COBRA, bisulfite sequencing, and MS-Pyroseq

Genomic DNA, 0.6 to 1 microgram, was bisulfite-converted using the EpiTect Bisulfite Kit (QIAGEN) according to the instructions of the manufacturer. Sequences including or adjacent to the index SNPs or Infinium CpG dinucleotides were amplified by PCR, using primers designed in MethPrimer [52]. PCR conditions, primer sequences, and corresponding unconverted genomic sequences are in Table S7. For COBRA we identified restriction sites in the converted sequences that differed according to methylation status of specific CpG dinucleotides and we digested the bisulfite PCR products with these enzymes followed by electrophoresis on 1.5% agarose gels. For bisulfite sequencing the PCR products were cloned using the TopoTA Cloning System (Invitrogen) and >12 plasmids sequenced for each gene in a given individual. MS-Pyroseq was performed by bisulfite converting genomic DNA samples, followed by PCR with gene-specific primers (designed in MethPrimer) and Pyrosequencing of the resulting PCR products at EpigenDx (Worcester, MA) using a Qiagen PSQ instrument. The methylation indices from MS-Pyroseq were calculated as the average percent methylation of ≥8 successive CpG dinucleotides between the primers (Table S7).

### Quantitative reverse transcription PCR (Q-PCR)

Q-PCR was performed using a 7300 Fast Real-Time PCR System (Applied Biosystems). Reactions were performed in triplicate in 96-well optical reaction plates. Each reaction contained cDNA reverse transcribed from 5 ng total RNA, 1X Power SYBR Green PCR master mix (Applied Biosystems) and 0.2 µM of each specific primer pair, which were designed using online D-Lux (Invitrogen) or Primer Express 3.0 software (Applied Biosystems). The thermal cycling conditions were primer annealing at 50°C for 2 min and an initial denaturation for 10 min at 95°C, followed by 40 cycles of 15 s at 95°C for denaturation and 1 min at 60°C for annealing and extension. The relative expression level of a target gene in a particular sample was calculated by the delta-CT method as described [53].

### Purification of NK cells and T-cells

NK cells were purified from human blood to >90% purity using immunomagnetic beads as previously described [54]. T-cells were isolated from blood of adults with DS and normal adult controls to >80% purity using a RosetteSep Kit (Sigma) according to the manufacturer's instructions.

## Supporting Information

Figure S1 Reliability of the Illumina Infinium data in technical replicates. In each graph the x- and y-axes indicate fractional methylation reported by the Infinium assays. A, technical replicates using PBL DNA from an adult with DS. B, technical replicates using DNA from a normal control. C, comparison of DS versus normal PBL. Correlation coefficients are indicated. For visual clarity, the methylation values for the loci listed in Table S2 are shown here. Similar correlation coefficients in technical replicates was found using data from all loci on the BeadArrays (not shown).
(0.07 MB PDF)

Figure S2 Bisulfite sequencing and MS-Pyroseq validating gain of methylation in the *CPT1B* promoter region in DS PBL. A, Bisulfite sequencing showing widespread loss of CpG methylation in broad regions spanning both ends of the large CGI. B, Map of the complex *CPT1B* promoter region, which contains several predicted alternative first exons. The CGI is represented by the white bar and the regions subjected to bisulfite sequencing are the grey bars. C, Result of MS-Pyroseq, showing percent methylation of a cluster of CpG dinucleotides (see primers Table S7) that are relatively hypermethylated in DS PBL.
(0.28 MB PDF)

Figure S3 Bisulfite sequencing validating gain of methylation in the *PLD6* first exon/CGI region in DS PBL. Bisulfite sequencing shows a strong and widespread gain of methylation in the CGI of *PLD6* in DS, affecting at least 22 contiguous CpGs. The CGI is represented by the white bar and the region subjected to bisulfite sequencing is the grey bar.
(0.10 MB PDF)

Figure S4 COBRA and bisulfite sequencing validating loss of methylation in the *TCF7* upstream region and gain of methylation in the CpG Island of *SH3BP2* in DS compared to normal PBL. A, COBRA for the *TCF7* upstream region showing less methylation in DS compared to normal PBL. The fractionated normal samples analyzed (PBMC, PMN, NKT, NK), and the complete blood counts (Table S5), indicate that the loss of methylation is not due to simple numerical changes in the percentages of mononuclear cells, PMNs, or NK cells (see main text for discussion). U, unmethylated; M, methylated; P, partially methylated. IVM, in vitro methylated DNA. B, COBRA for the *SH3BP2* CGI region showing increased methylation in DS compared to normal PBL, albeit with variability among the cases (compare with large scale MS-Pyroseq in Figure 3 of the main text). Normal fractionated mononuclear cells, PMNs and NK cells do not show the abnormal pattern of methylation seen in DS PBL. C, Bisulfite sequencing showing that the loss of methylation in the upstream region of *TCF7* in DS affects at least 8 contiguous CpGs. This CG-rich region is well conserved across vertebrate species, comparable to exon conservation (Multiz Alignment and Conservation track at http://genome.ucsc.edu). D, Bisulfite sequencing showing that the gain of methylation in the CGI region of *SH3BP2* in DS affects at least 10 contiguous CpGs. E, F, maps of the *TCF7* upstream region and CGI and the *SH3BP2* CGI region, respectively, showing the areas analyzed by bisulfite sequencing/COBRA (grey boxes).
(0.19 MB PDF)

Figure S5 MS-Pyroseq confirming altered CpG methylation in T-cells from DS versus normal controls. The maps show the regions subjected to MS-Pyroseq (long grey bars) and the locations of the index SNP for *TMEM131* and the most informative Infinium probes for *SUMO3*, *SH3BP2* and *CD3Z* (small grey squares).
(0.06 MB PDF)

Figure S6 Isoform-specificity of the epigenetic lesion affecting *TMEM131*: lack of differential methylation in the upstream promoter-associated CGI and lack of differential expression of the long isoform of *TMEM131* mRNA. A, Expression of the long isoform mRNA measured by Q-PCR shows no difference between DS and normal PBL. B, bisulfite sequencing of the CGI corresponding to the promoter region of the long mRNA isoform; very little methylation is seen in both DS and normal PBL. C, Map of the *TMEM131* genomic locus showing the initiation sites for the long and short mRNA isoforms. Differential CpG methylation is present in the immediate upstream region of the short isoform, which is differentially expressed at the mRNA level (see Figure 3 and Figure 4 in main text).
(0.19 MB PDF)

Figure S7 Increased methylation of the *TCF7* CGI in DS compared to normal PBL. The percent methylation was determined by MS-Pyroseq using primers described in Table S7. The CGI is indicated by the open rectangle and the region examined by MS-Pyroseq is shown by the grey rectangle under the map.
(0.06 MB PDF)

Table S1 Differentially methylated loci from the MSNP screen. Loci with increased methylation in DS are in red and loci with decreased methylation in DS are in blue (ANOVA p<.01; ≥1.2 fold difference in methylation index; only unique cases included in the ANOVA.). The methylation indices were calculated from the preservation of intensity in the +*Hpa*II genomic representations (see Materials and Methods).
(0.07 MB PDF)

Table S2 Differentially methylated loci from the Illumina Infinium screen using DNA from PBL. Loci with increased methylation in DS are in red and loci with decreased methylation in DS are in blue (ANOVA p<.01; >1.2 fold difference in methylation index and >0.1 absolute difference in methylation index; only unique cases included in the ANOVA.) Methylation indices are the fraction of signal intensity from methylated CpG, as determined in the Infinium assays, which compare C to T signals after bisulfite conversion. Coefficients of the logistic regression fit, which represent size of the contribution of each predictor to classification status, and their corresponding odds ratios, are shown in the last 2 columns.
(0.06 MB PDF)

Table S3 Stability of DNA methylation over time in adults with DS.
(0.05 MB PDF)

Table S4 Methylation values in cases of DS with mosaicism.
(0.06 MB PDF)

Table S5 Complete blood counts in 4 adults with DS. The normal ranges for Columbia University Medical Center are indicated.
(0.06 MB PDF)

Table S6 Differentially methylated loci from the Illumina Infinium screen using DNA from purified T-cells. Loci with increased methylation in DS are in red and loci with decreased methylation in DS are in blue. Statistical criteria were ANOVA p<.01; >1.3 fold difference in methylation index and >0.15 absolute difference in methylation index between DS T-cells and normal T-cells. One further example, the *CD3Z* gene, was also differentially methylated both in total PBL and in T-cells, as indicated by the MS-Pyroseq data (Figure S5), but as the methylation values were all in a very low range (2 - 14% methylation) the MS-Pyroseq was more senstive than the Infinium assay for detecting the difference. One gene, *ALX4*, which is queried by the Infinium BeadChip at two different CpG dinucleotides separated by 5.5 kb, showed opposite changes in fractional methylation at these two positions.
(0.07 MB PDF)

Table S7 PCR primers used in this study.
(0.05 MB PDF)
