# Supplementary figures and images for: Altered DNA Methylation in Leukocytes with Trisomy 21
Source: PLoS Genet. 2010 Nov 18;6(11):e1001212. doi: 10.1371/journal.pgen.1001212 (PMC2987931; doi:10.1371/journal.pgen.1001212)

Suppl. Figure S1

A.

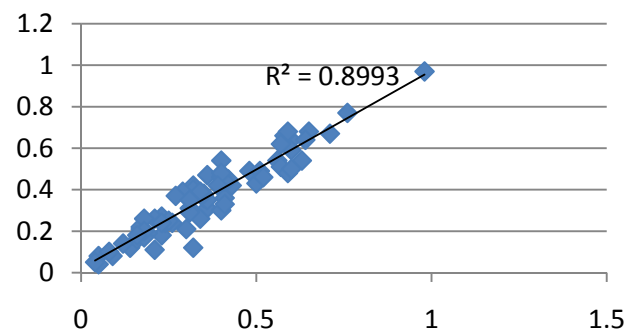

X: DS702-A  
Y: DS702-B

B.

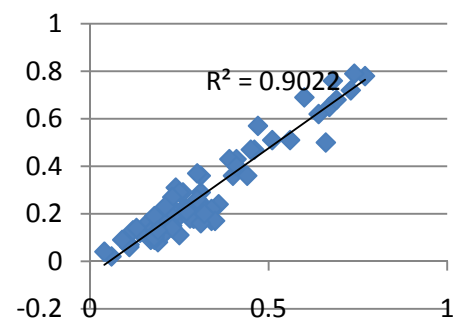

X: normal control 5194-A  
Y: normal control 5194-B

C.

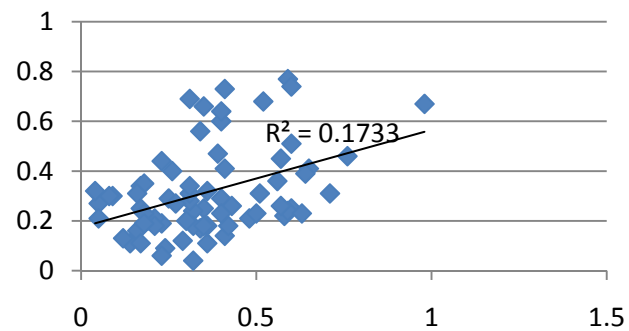

X: DS702-A  
Y: normal control 5194-A

Supplement: Figure S1 — Reliability of the Illumina Infinium data in technical replicates. In each graph the x- and y-axes indicate fractional methylation reported by the Infinium assays. A, technical replicates using PBL DNA from an adult with DS. B, technical replicates using DNA from a normal control. C, comparison of DS versus normal PBL. Correlation coefficients are indicated. For visual clarity, the methylation values for the loci listed in Table S2 are shown here. Similar correlation coefficients in technical replicates was found using data from all loci on the BeadArrays (not shown). (0.07 MB PDF) [file pgen.1001212.s001.pdf]

Suppl. Figure S2

A.

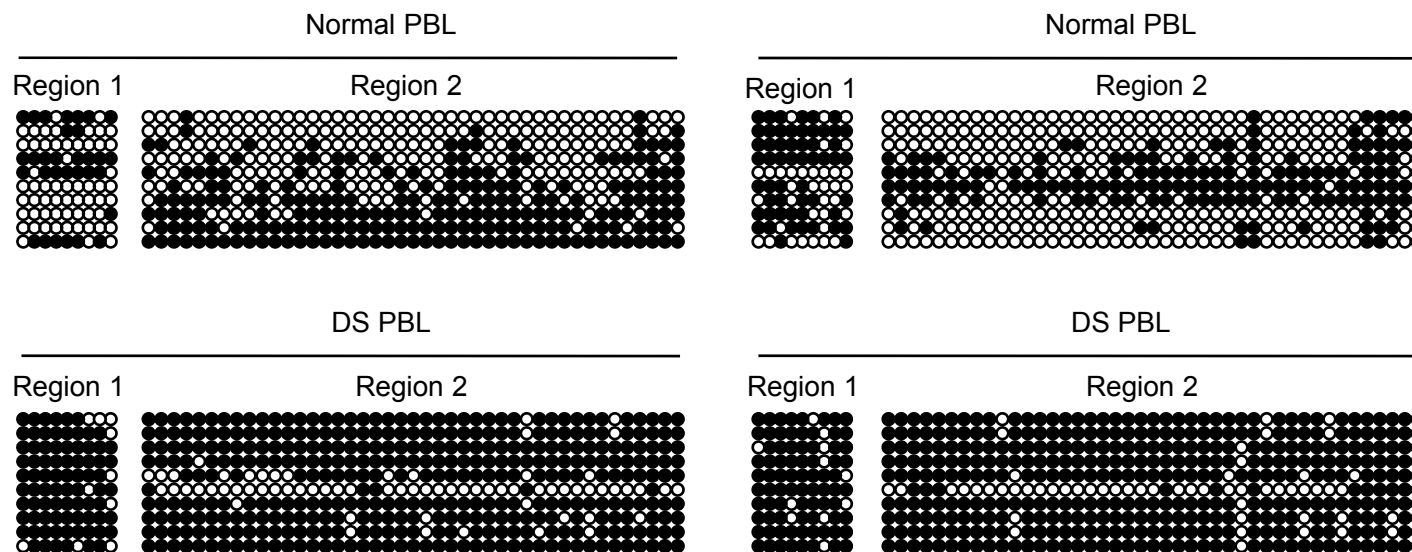

B.

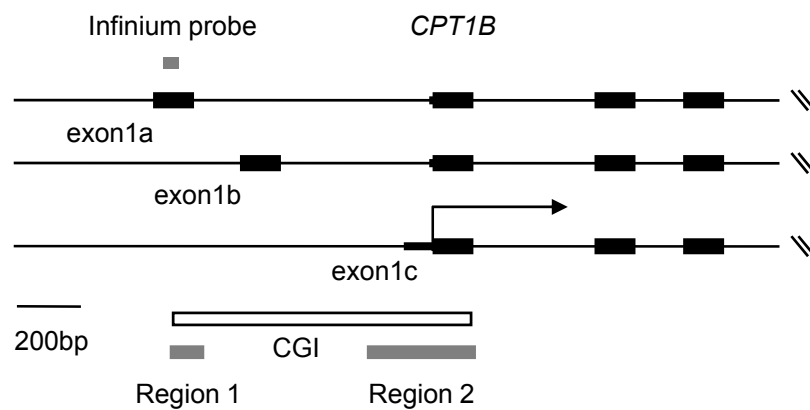

C.

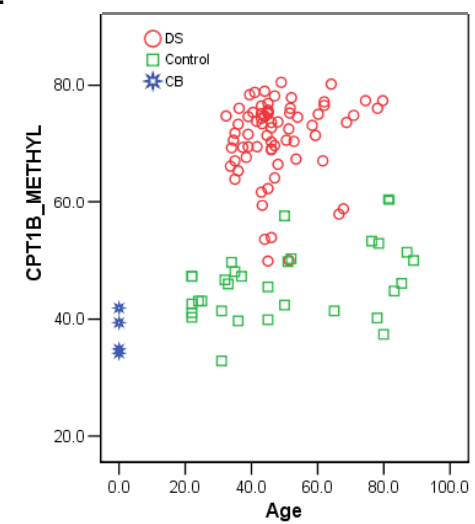

Supplement: Figure S2 — Bisulfite sequencing and MS-Pyroseq validating gain of methylation in the CPT1B promoter region in DS PBL. A, Bisulfite sequencing showing widespread loss of CpG methylation in broad regions spanning both ends of the large CGI. B, Map of the complex CPT1B promoter region, which contains several predicted alternative first exons. The CGI is represented by the white bar and the regions subjected to bisulfite sequencing are the grey bars. C, Result of MS-Pyroseq, showing percent methylation of a cluster of CpG dinucleotides (see primers Table S7) that are relatively hypermethylated in DS PBL. (0.28 MB PDF) [file pgen.1001212.s002.pdf]

Suppl. Figure S3

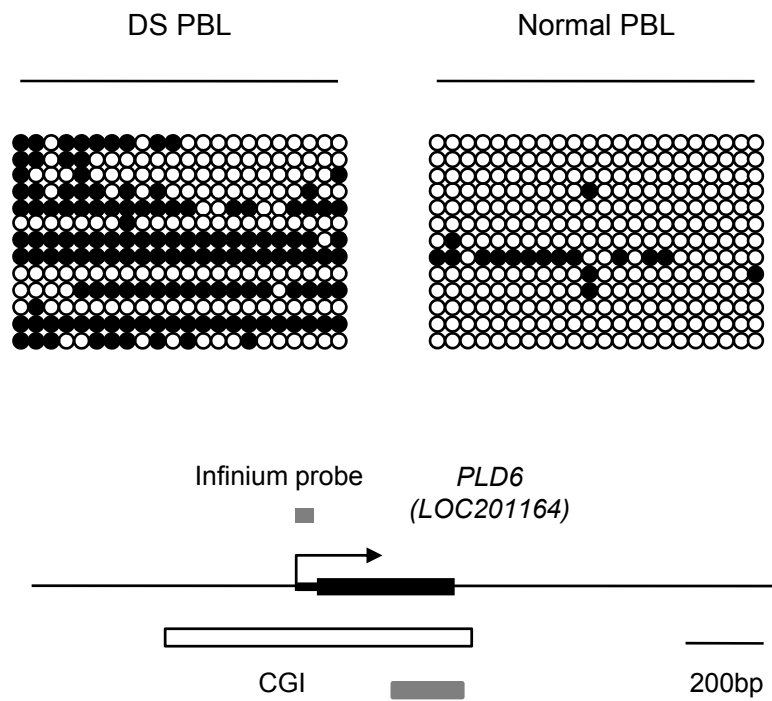

Supplement: Figure S3 — Bisulfite sequencing validating gain of methylation in the PLD6 first exon/CGI region in DS PBL. Bisulfite sequencing shows a strong and widespread gain of methylation in the CGI of PLD6 in DS, affecting at least 22 contiguous CpGs. The CGI is represented by the white bar and the region subjected to bisulfite sequencing is the grey bar. (0.10 MB PDF) [file pgen.1001212.s003.pdf]

Suppl. Figure S4

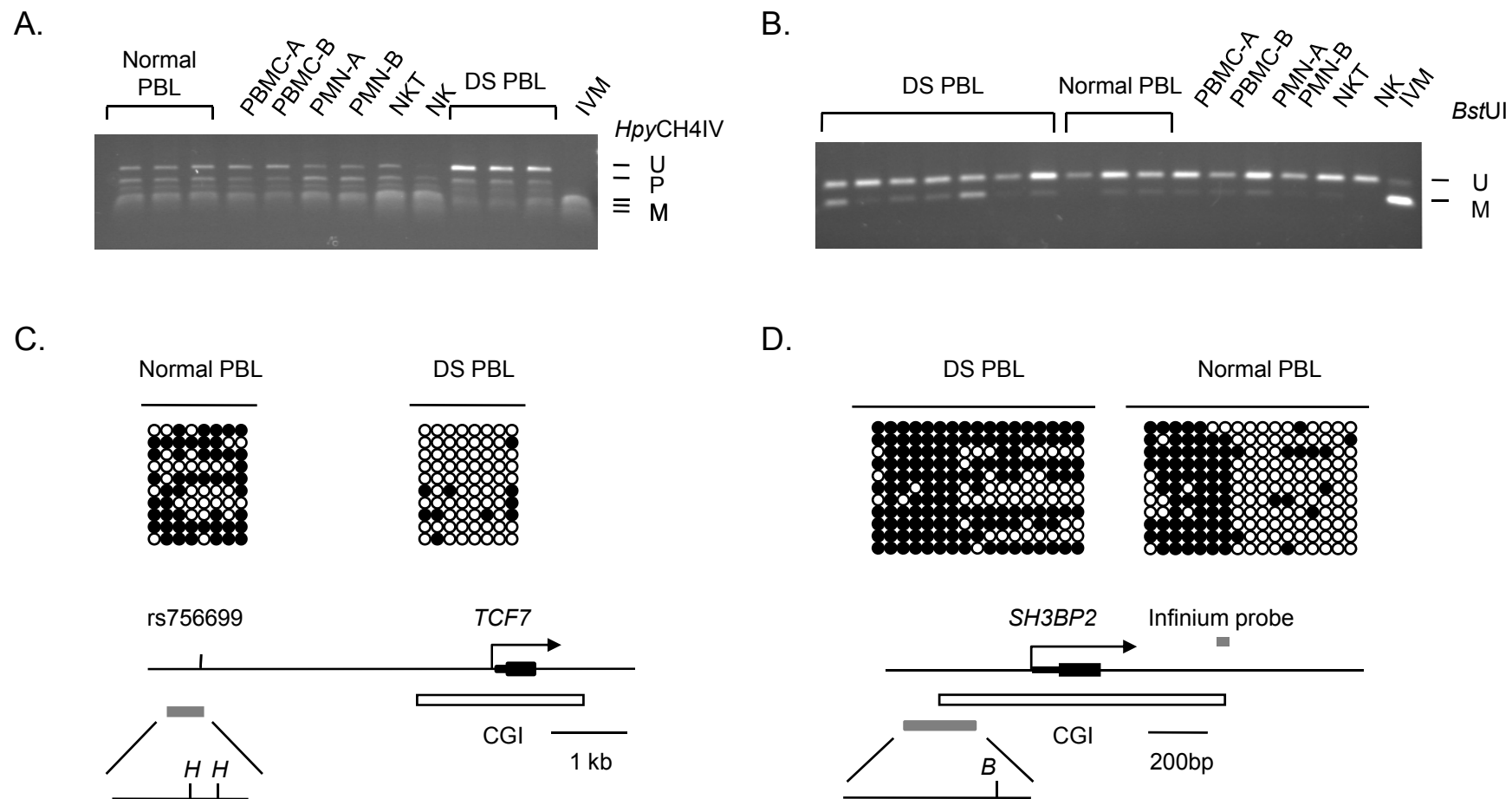

Supplement: Figure S4 — COBRA and bisulfite sequencing validating loss of methylation in the TCF7 upstream region and gain of methylation in the CpG Island of SH3BP2 in DS compared to normal PBL. A, COBRA for the TCF7 upstream region showing less methylation in DS compared to normal PBL. The fractionated normal samples analyzed (PBMC, PMN, NKT, NK), and the complete blood counts (Table S5), indicate that the loss of methylation is not due to simple numerical changes in the percentages of mononuclear cells, PMNs, or NK cells (see main text for discussion). U, unmethylated; M, methylated; P, partially methylated. IVM, in vitro methylated DNA. B, COBRA for the SH3BP2 CGI region showing increased methylation in DS compared to normal PBL, albeit with variability among the cases (compare with large scale MS-Pyroseq in Figure 3 of the main text). Normal fractionated mononuclear cells, PMNs and NK cells do not show the abnormal pattern of methylation seen in DS PBL. C, Bisulfite sequencing showing that the loss of methylation in the upstream region of TCF7 in DS affects at least 8 contiguous CpGs. This CG-rich region is well conserved across vertebrate species, comparable to exon conservation (Multiz Alignment and Conservation track at http://genome.ucsc.edu). D, Bisulfite sequencing showing that the gain of methylation in the CGI region of SH3BP2 in DS affects at least 10 contiguous CpGs. E, F, maps of the TCF7 upstream region and CGI and the SH3BP2 CGI region, respectively, showing the areas analyzed by bisulfite sequencing/COBRA (grey boxes). (0.19 MB PDF) [file pgen.1001212.s004.pdf]

Suppl. Figure S5

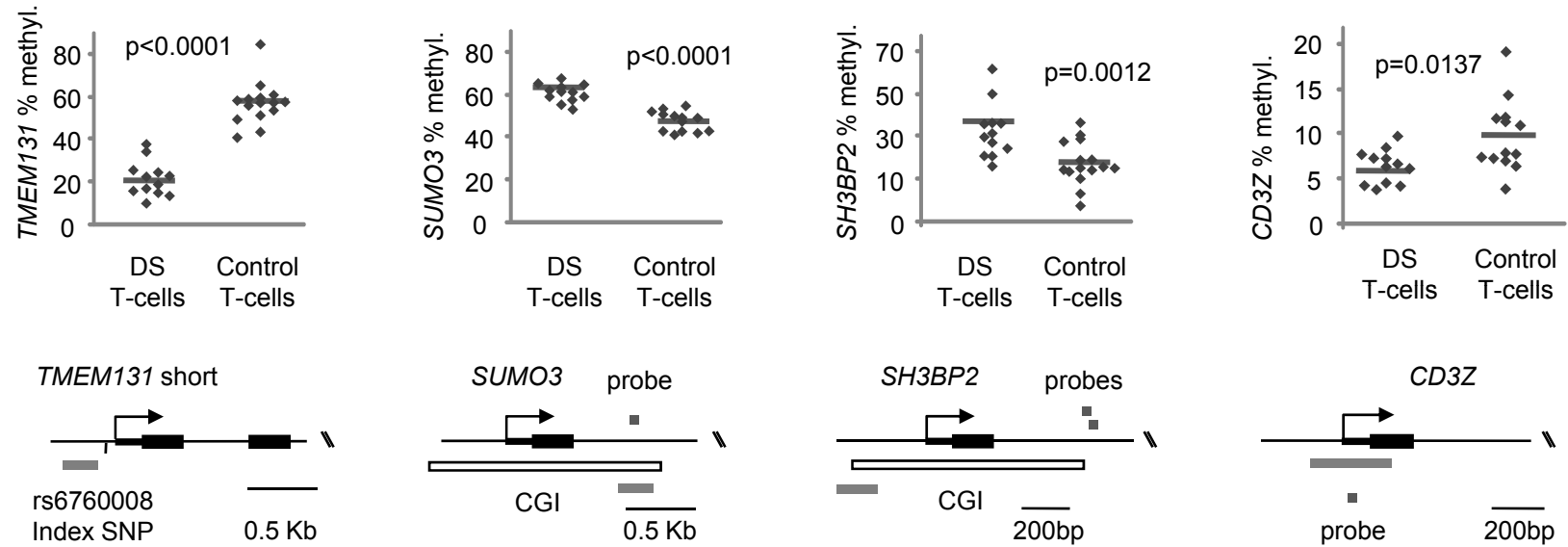

Supplement: Figure S5 — MS-Pyroseq confirming altered CpG methylation in T-cells from DS versus normal controls. The maps show the regions subjected to MS-Pyroseq (long grey bars) and the locations of the index SNP for TMEM131 and the most informative Infinium probes for SUMO3, SH3BP2 and CD3Z (small grey squares). (0.06 MB PDF) [file pgen.1001212.s005.pdf]

Suppl. Figure S6

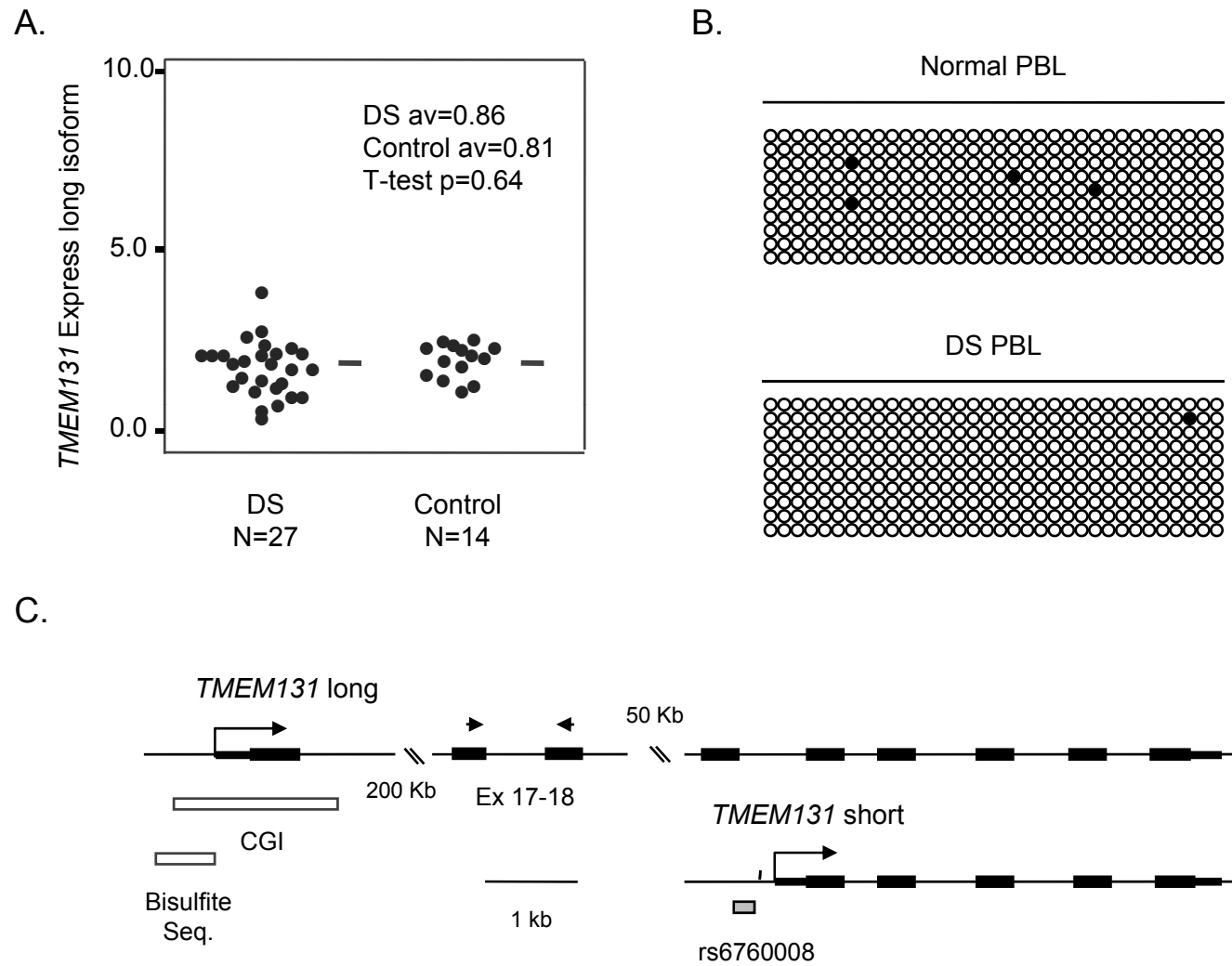

Supplement: Figure S6 — Isoform-specificity of the epigenetic lesion affecting TMEM131: lack of differential methylation in the upstream promoter-associated CGI and lack of differential expression of the long isoform of TMEM131 mRNA. A, Expression of the long isoform mRNA measured by Q-PCR shows no difference between DS and normal PBL. B, bisulfite sequencing of the CGI corresponding to the promoter region of the long mRNA isoform; very little methylation is seen in both DS and normal PBL. C, Map of the TMEM131 genomic locus showing the initiation sites for the long and short mRNA isoforms. Differential CpG methylation is present in the immediate upstream region of the short isoform, which is differentially expressed at the mRNA level (see Figure 3 and Figure 4 in main text). (0.19 MB PDF) [file pgen.1001212.s006.pdf]

Suppl. Figure S7

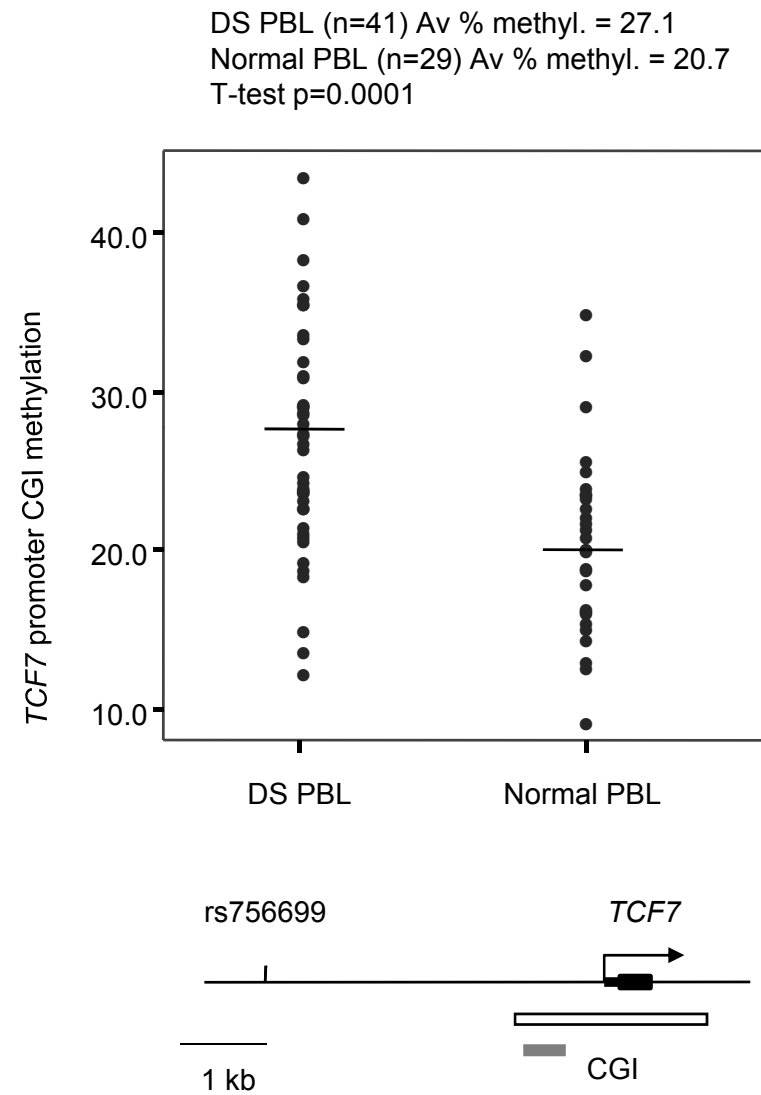

Supplement: Figure S7 — Increased methylation of the TCF7 CGI in DS compared to normal PBL. The percent methylation was determined by MS-Pyroseq using primers described in Table S7. The CGI is indicated by the open rectangle and the region examined by MS-Pyroseq is shown by the grey rectangle under the map. (0.06 MB PDF) [file pgen.1001212.s007.pdf]
