# Supplementary material for: Altered DNA Methylation in Leukocytes with Trisomy 21
Source: PLoS Genet. 2010 Nov 18;6(11):e1001212. doi: 10.1371/journal.pgen.1001212 (PMC2987931; doi:10.1371/journal.pgen.1001212)
Supplement: Table S1 — Differentially methylated loci from the MSNP screen. Loci with increased methylation in DS are in red and loci with decreased methylation in DS are in blue (ANOVA p<.01; ≥1.2 fold difference in methylation index; only unique cases included in the ANOVA.). The methylation indices were calculated from the preservation of intensity in the +HpaII genomic representations (see Materials and Methods). (0.07 MB PDF) [file pgen.1001212.s008.pdf]

probe set	dbSNP RS ID	Chr	Cytoband	Associated Gene(s)	Product(s)	SNP Position Relative to Gene(s)	DS PBL av	Control PBL av	DS / Control	DS - Control
SNP_A-2305831	rs11863621	16	q23.3	CDH13	member of the cadherin superfamily	intergenic	0.722	0.298571429	2.41818182	0.423428571
SNP_A-1974529	rs1720830	3	q22.3	FAM62C	family with sequence similarity 62 member C	promoter CGI	0.732	0.32	2.2875	0.412
SNP_A-1785577	rs2654974	15	q26.3	LOC145814		intron	0.862	0.391428571	2.20218978	0.470571429
SNP_A-4249213	rs2294309	6	p22.1	ZNF184	zinc finger protein	upstream	0.522	0.24	2.175	0.282
SNP_A-2104584	rs360529	20	p12.1	SPTLC3	serine palmitoyltransferase	intron	0.452	0.221428571	2.04129032	0.230571429
SNP_A-4287008	rs9318544	13	q31.1	POU4F1	class IV POU domain-containing transcription factor	intergenic	0.894	0.467142857	1.91376147	0.426857143
SNP_A-1877369	rs16941688	18	p11.32	----		intergenic	0.276	0.145714286	1.89411765	0.130285714
SNP_A-1953165	rs11651551	17	q25.1	SDK2	member of the immunoglobulin superfamily	upstream	0.386	0.208571429	1.85068493	0.177428571
SNP_A-1870762	rs2167594	15	q21.1	B2M /// TRIM69	beta-2-microglobulin//RING-B-box-coiled-coil family	downstream /// upstream	0.148	0.082857143	1.7862069	0.065142857
SNP_A-2313951	rs7539505	1	q24.3	KIAA0859		downstream	0.842	0.485714286	1.73352941	0.356285714
SNP_A-2111041	rs7552818	1	q42.13	WNT9A	member of the WNT gene family	downstream	0.628	0.377142857	1.66515152	0.250857143
SNP_A-2222429	rs17012263	2	q14.2	C1QL2	complement component 1, q subcomponent-like 2	downstream	0.628	0.381428571	1.64644195	0.246571429
SNP_A-1885541	rs6558962	8	p23.2	CSMD1	CUB and sushi domain-containing protein	intergenic	0.536	0.33	1.62424242	0.206
SNP_A-2294769	rs8011143	14	q11.2	ABHD4	abhydrolase domain containing 4	promoter CGI; intron 1	0.108	0.067142857	1.60851064	0.040857143
SNP_A-1812148	rs4413548	5	q35.3	PROP1	PROP paired-like homeobox 1	downstream	0.682	0.428571429	1.59133333	0.253428571
SNP_A-1994721	rs719213	8	q23.3	CSMD3	CUB and sushi domain-containing protein	upstream	0.184	0.115714286	1.59012346	0.068285714
SNP_A-1983012	rs898355	5	q22.3	KCNN2	potassium intermediate/small conductance calcium-activate	intron	0.498	0.317142857	1.57027027	0.180857143
SNP_A-2200145	rs17207640	22	q11.21	TUBA8 /// USP18	tubulin, alpha 8//ubiquitin specific peptidase 18	promoter CGI /// downstream	0.194	0.125714286	1.54318182	0.068285714
SNP_A-2003622	rs909077	14	q11.2	TCRA	T-cell receptor alpha chain	intron	0.816	0.537142857	1.51914894	0.278857143
SNP_A-2149238	rs4959782	6	p25.2	TUBB2B /// PSMG4	beta isoform of tubulin//roteasome assembly chaperone	upstream /// upstream	0.18	0.118571429	1.51807229	0.061428571
SNP_A-2079967	rs12533831	7	q36.1	GBX1 /// ASB10	gastrulation brain homeobox//ankyrin repeat and SOCS box-	upstream /// downstream	0.56	0.37	1.51351351	0.19
SNP_A-2245624	rs304350	4	q32.3	----		intergenic	0.44	0.294285714	1.49514563	0.145714286
SNP_A-2061534	rs6467723	7	q33	CREB3L2	cAMP responsive element binding protein	intron	0.41	0.274285714	1.49479167	0.135714286
SNP_A-1792622	rs4949308	1	p35.2	LAPTM5 /// SDC3	lysosomal protein transmembrane 5//syndecan proteoglycar	intergenic	1.436	0.97	1.48041237	0.466
SNP_A-2168378	rs1550862	2	p21	----		intergenic	0.782	0.528571429	1.47945946	0.253428571
SNP_A-2267500	rs1966618	3	q29	BDH1	short-chain dehydrogenase/reductase gene family	promoter CGI; intron 2	0.486	0.331428571	1.46637931	0.154571429
SNP_A-4276450	rs10002498	4	p12	NFXL1 /// CNGA1	nuclear transcription factor, X-box binding-like 1//protein inv	upstream /// downstream	0.314	0.218571429	1.43660131	0.095428571
SNP_A-1808274	rs11134037	5	p15.33	///		intergenic	0.746	0.527142857	1.41517615	0.218857143
SNP_A-2249260	rs1146297	1	p12	MAN1A2	member of family 38 of the glycosyl hydrolases	promoter CGI	0.348	0.247142857	1.40809249	0.100857143
SNP_A-2312342	rs11648858	16	q24.1	COX4NB	neighbor of COX4	intron	0.36	0.255714286	1.40782123	0.104285714
SNP_A-1891502	rs2290526	8	q12.1	TOX	thymocyte selection-associated high mobility group box prot	promoter CGI	0.372	0.264285714	1.40756757	0.107714286
SNP_A-2039835	rs2048253	3	q27.2	C3orf70 /// EHHADH	hypothetical protein LOC285382 //enzyme in peroxisomal b	upstream /// downstream	0.91	0.647142857	1.40618102	0.262857143
SNP_A-2206655	rs924041	6	q27	----		intergenic	0.764	0.545714286	1.4	0.218285714
SNP_A-4243924	rs17057654	3	p14.3	ARHGEF3	Rho guanine nucleotide exchange factor	intron	0.636	0.462857143	1.37407407	0.173142857
SNP_A-1907500	rs1014422	15	q26.2	----		intergenic	1.056	0.768571429	1.3739777	0.287428571
SNP_A-4271635	rs12218684	10	q26.2	----		intergenic	0.88	0.644285714	1.36585366	0.235714286
SNP_A-2147411	rs11145647	9	q21.2	GNAQ	guanine nucleotide-binding protein	intron	0.774	0.57	1.35789474	0.204
SNP_A-1862688	rs11766104	7	p21.1	AHR	aryl hydrocarbon receptor	intergenic	0.88	0.655714286	1.34204793	0.224285714
SNP_A-2133790	rs6713219	2	q23.3	STAM2	signal transducing adaptor molecule (SH3 domain and ITAM	upstream	0.732	0.547142857	1.33785901	0.184857143

SNP_A-2079187	rs16978757	19	p13.13	KLF1	Kruppel-like factor 1	exon 1; near promoter CGI	0.468	0.35	1.33714286	0.118
SNP_A-4296997	rs16836497	4	p16.2	CYTL1	cytokine-like protein	intergenic	0.93	0.698571429	1.33128834	0.231428571
SNP_A-2266761	rs10849538	12	p13.31	LRRC23	leucine rich repeat containing 23	intron	0.508	0.382857143	1.32686567	0.125142857
SNP_A-2021059	rs2887763	11	q24.1	----		intergenic	0.248	0.187142857	1.32519084	0.060857143
SNP_A-1840668	rs16947926	17	p12	HS3ST3A1	heparan sulfate (glucosamine) 3-O-sulfotransferase 3A1	downstream	1.336	1.01	1.32277228	0.326
SNP_A-1994007	rs16907621	8	q21.13	----		intergenic	1.106	0.837142857	1.32116041	0.268857143
SNP_A-1960078	rs12023763	1	q23.3	----		intergenic	1.302	0.987142857	1.31895803	0.314857143
SNP_A-2212570	rs8192650	8	q24.13	ZHX2	zinc fingers and homeoboxes gene family	upstream	1.07	0.812857143	1.31634446	0.257142857
SNP_A-2064773	rs17496910	4	p15.31	----		intergenic	0.956	0.727142857	1.31473477	0.228857143
SNP_A-1894207	rs3019442	6	q26	PARK2	component of a multiprotein E3 ubiquitin ligase complex, me	intron	0.632	0.482857143	1.30887574	0.149142857
SNP_A-2211111	rs2185384	1	q32.2	KCNH1	potassium voltage-gated channel, subfamily H, member 1	intron	0.776	0.594285714	1.30576923	0.181714286
SNP_A-2145267	rs10967965	9	p21.2	MOBKL2B		intron	1.262	0.974285714	1.29530792	0.287714286
SNP_A-1821379	rs2187431	18	q21.1	ZBTB7C	zinc finger and BTB domain containing 7C	intron	0.784	0.622857143	1.2587156	0.161142857
SNP_A-1938991	rs7097422	10	p12.1	KIAA1217		intron	0.99	0.815714286	1.21366025	0.174285714
SNP_A-2031660	rs4766772	12	q24.21	----		intergenic	0.57	0.69	0.82608696	-0.12
SNP_A-2120497	rs3947662	1	p36.32	AJAP1	adherens junctions associated protein 1	intron	0.542	0.711428571	0.76184739	-0.16942857
SNP_A-2175482	rs11855819	15	q14	TMCO5A	transmembrane and coiled-coil domains 5A	upstream	0.476	0.638571429	0.74541387	-0.16257143
SNP_A-4287344	rs17164354	5	p15.2	----		intergenic	0.638	0.901428571	0.70776545	-0.26342857
SNP_A-1929546	rs17068501	13	q14.13	LRCH1	leucine-rich repeats and calponin homology (CH) domain cor	intron	0.6	0.848571429	0.70707071	-0.24857143
SNP_A-4286506	rs12911169	15	q13.1	KIAA0574		intron	0.634	0.9	0.70444444	-0.266
SNP_A-4304825	rs2763948	13	q32.3	CLYBL	citrate lyase beta like	intron	0.526	0.754285714	0.69734848	-0.22828571
SNP_A-4270660	rs2839419	21	q22.3	C2CD2	C2 calcium-dependent domain containing 2	intron	0.316	0.461428571	0.68482972	-0.14542857
SNP_A-4270423	rs7283121	21	q22.11	MRAP	melanocortin 2 receptor accessory protein	intron	0.378	0.555714286	0.68020566	-0.17771429
SNP_A-1909701	rs4806371	19	q12	----		intergenic	0.49	0.737142857	0.66472868	-0.24714286
SNP_A-2008183	rs7113375	11	p15.1	SAA2	serum amyloid A2	intron	0.454	0.697142857	0.65122951	-0.24314286
SNP_A-2179466	rs756699	5	q31.1	TCF7	transcription factor 7 (T-cell specific, HMG-box)	upstream	0.386	0.597142857	0.64641148	-0.21114286
SNP_A-2183520	rs1947384	8	p21.1	C8orf80	hypothetical protein LOC389643	intron	0.608	0.968571429	0.62772861	-0.36057143
SNP_A-2066201	rs2909985	7	q11.22	CALN1	similarity to the calcium-binding proteins of the calmodulin f	intron	0.406	0.655714286	0.61917211	-0.24971429
SNP_A-2083869	rs114873	12	q21.33	----		intergenic	0.42	0.71	0.5915493	-0.29
SNP_A-1870078	rs4328485	17	q24.3	KCNJ2	potassium inwardly-rectifying channel, subfamily J, member	downstream	0.498	0.965714286	0.51568047	-0.46771429
SNP_A-2136365	rs6760008	2	q11.2	TMEM131	transmembrane protein 131	intron/alt promoter	0.3	0.671428571	0.44680851	-0.37142857
