# Supplementary material for: Altered DNA Methylation in Leukocytes with Trisomy 21
Source: PLoS Genet. 2010 Nov 18;6(11):e1001212. doi: 10.1371/journal.pgen.1001212 (PMC2987931; doi:10.1371/journal.pgen.1001212)
Supplement: Table S2 — Differentially methylated loci from the Illumina Infinium screen using DNA from PBL. Loci with increased methylation in DS are in red and loci with decreased methylation in DS are in blue (ANOVA p<.01; >1.2 fold difference in methylation index and >0.1 absolute difference in methylation index; only unique cases included in the ANOVA.) Methylation indices are the fraction of signal intensity from methylated CpG, as determined in the Infinium assays, which compare C to T signals after bisulfite conversion. Coefficients of the logistic regression fit, which represent size of the contribution of each predictor to classification status, and their corresponding odds ratios, are shown in the last 2 columns. (0.06 MB PDF) [file pgen.1001212.s009.pdf]

probe set	Chr	MapInfo	Symbol	Product	Av DS	Av Control	Av DS / Av Control	Av DS - Av Control	O.R.	Coefficient
cg05590257	17	17050295	PLD6_LOC201164	hypothetical protein LOC201164	0.35	0.12	2.93	0.23	0.00E+00	-2.15E+01
cg01351032	16	10878692	CIITA	class II transactivator	0.21	0.08	2.67	0.13	0.00E+00	-1.50E+01
cg20727114	19	54885932	CPT1C	carnitine palmitoyltransferase 1C	0.30	0.11	2.61	0.18	0.00E+00	-1.93E+01
cg15633390	4	100070234	EIF4E	eukaryotic translation initiation factor 4E	0.32	0.12	2.61	0.20	0.00E+00	-1.66E+01
cg00230502	12	118256764	CCDC60	hypothetical protein LOC160777	0.22	0.09	2.60	0.14	0.00E+00	-1.90E+01
cg14859460	5	178354850	GRM6	glutamate receptor; metabotropic 6 precursor	0.32	0.12	2.55	0.19	0.00E+00	-1.16E+01
cg08822227	4	2790266	SH3BP2	SH3-domain binding protein 2	0.45	0.18	2.45	0.26	0.00E+00	-1.07E+01
cg14972143	4	100070026	EIF4E	eukaryotic translation initiation factor 4E	0.30	0.13	2.39	0.17	0.00E+00	-1.81E+01
cg02880176	17	17050542	PLD6_LOC201164	hypothetical protein LOC201164	0.23	0.10	2.34	0.13	0.00E+00	-1.52E+01
cg14159672	1	204085802	FLJ32569	hypothetical protein LOC148811	0.46	0.20	2.33	0.26	2.00E-04	-8.73E+00
cg13105904	14	23969884	KIAA0323	hypothetical protein LOC23351	0.39	0.18	2.15	0.21	2.90E-02	-3.54E+00
cg13906813	6	33148892	HLA-DPA1	major histocompatibility complex; class II; DP alpl	0.43	0.21	2.08	0.22	0.00E+00	-1.47E+01
cg08832227	12	4890954	KCNA1	potassium voltage-gated channel; shaker-related	0.41	0.21	1.96	0.20	0.00E+00	-2.92E+01
cg00983520	22	49363933	CPT1B	carnitine palmitoyltransferase 1B isoform a	0.57	0.29	1.93	0.27	0.00E+00	-1.27E+01
cg07991621	4	2790277	SH3BP2	SH3-domain binding protein 2	0.49	0.26	1.93	0.24	5.00E-04	-7.61E+00
cg22730004	1	156923342	SPTA1	spectrin; alpha; erythrocytic 1 (elliptocytosis 2)	0.37	0.20	1.92	0.18	0.00E+00	-1.14E+01
cg11507178	11	118286725	BCL9L	B-cell CLL/lymphoma 9-like	0.40	0.21	1.88	0.19	0.00E+00	-1.46E+01
cg10557828	3	147362332	PLOD2	procollagen-lysine; 2-oxoglutarate 5-dioxygenase	0.35	0.19	1.85	0.16	0.00E+00	-1.06E+01
cg00660989	14	44502266	BTBD5	BTB (POZ) domain containing 5	0.53	0.29	1.84	0.24	5.00E-04	-7.68E+00
cg20974196	19	810983	CFD	complement factor D preproprotein	0.31	0.17	1.79	0.14	0.00E+00	-2.43E+01
cg12448933	17	70178973	RAB37	RAB37; member RAS oncogene family isoform 1	0.23	0.13	1.78	0.10	0.00E+00	-2.58E+01
cg21053323	21	45061813	SUMO3	small ubiquitin-like modifier protein 3	0.44	0.25	1.77	0.19	0.00E+00	-1.47E+01
cg15439196	10	104666897	CNNM2	cyclin M2 isoform 3	0.35	0.20	1.70	0.14	0.00E+00	-3.79E+01
cg14917512	19	3045685	GNA11	guanine nucleotide binding protein (G protein); al	0.30	0.18	1.69	0.12	0.00E+00	-2.12E+01
cg14893161	1	204085874	FLJ32569	hypothetical protein LOC148811	0.56	0.33	1.69	0.23	0.00E+00	-1.10E+01
cg13749822	4	145786113	HHIP	hedgehog-interacting protein	0.48	0.28	1.68	0.19	0.00E+00	-2.94E+01
cg04739570	3	128831301	PODXL2	endoglycan	0.39	0.23	1.67	0.16	0.00E+00	-1.35E+01
cg02498063	12	118256960	CCDC60	hypothetical protein LOC160777	0.65	0.39	1.66	0.26	0.00E+00	-1.41E+01
cg05822532	7	73080467	ELN	elastin	0.51	0.31	1.64	0.20	6.00E-04	-7.44E+00
cg21376883	1	234916855	ACTN2	actinin; alpha 2	0.27	0.17	1.64	0.11	0.00E+00	-3.08E+01
cg15642326	19	50445986	MARK4	MAP/microtubule affinity-regulating kinase 4	0.33	0.20	1.63	0.13	0.00E+00	-2.14E+01
cg26711820	12	79625820	MYF6	myogenic factor 6 (herculin)	0.26	0.16	1.61	0.10	0.00E+00	-4.68E+01
cg05968233	19	6323277	ALKBH7	spermatogenesis associated 11	0.33	0.21	1.60	0.12	0.00E+00	-1.18E+01
cg22341310	19	52740395	ZNF541	zinc finger protein 541	0.28	0.18	1.57	0.10	0.00E+00	-1.64E+01
cg09697795	19	55358191	SCRL	hypothetical protein LOC126123	0.28	0.18	1.57	0.10	0.00E+00	-3.24E+01
cg06896207	19	2046364	MOBKL2A	MOB-LAK	0.43	0.27	1.56	0.15	0.00E+00	-2.52E+01
cg03454353	9	114859185	ZFP37	zinc finger protein 37 homolog	0.34	0.22	1.56	0.12	0.00E+00	-2.20E+01
cg07612655	20	47618924	PTGIS	prostaglandin I2 (prostacyclin) synthase	0.44	0.28	1.56	0.16	0.00E+00	-2.22E+01

cg16907514	14	55115851	KTN1	kinectin 1	0.42	0.27	1.56	0.15	3.00E-04	-8.28E+00
cg16786458	5	149089013	PPARGC1B	PGC-1-related estrogen receptor alpha coactivato	0.53	0.34	1.53	0.18	0.00E+00	-2.36E+01
cg11004890	20	3166500	SLC4A11	solute carrier family 4 member 11	0.64	0.43	1.51	0.22	1.00E-04	-9.40E+00
cg16334795	21	41460764	BACE2	beta-site APP-cleaving enzyme 2 isoform A prepr	0.44	0.29	1.51	0.15	0.00E+00	-1.92E+01
cg08431931	22	40724605	MGC26816	hypothetical protein LOC164684	0.42	0.28	1.50	0.14	1.39E-02	-4.28E+00
cg22919728	3	127725180	CHST13	carbohydrate (chondroitin 4) sulfotransferase 13	0.52	0.34	1.50	0.17	2.00E-04	-8.45E+00
cg22165685	10	134901282	VENTX	VENT homeobox	0.45	0.30	1.49	0.15	0.00E+00	-1.49E+01
cg04156850	17	70914167	GRB2	growth factor receptor-bound protein 2 isoform 1	0.33	0.22	1.48	0.11	0.00E+00	-2.71E+01
cg15804973	6	137156206	MAP3K5	mitogen-activated protein kinase kinase kinase 5	0.38	0.25	1.48	0.12	2.20E-03	-6.14E+00
cg01405107	17	44026634	HOXB5	homeo box B5	0.51	0.34	1.48	0.16	0.00E+00	-1.62E+01
cg06500079	17	565783	VPS53	VPS53 protein	0.37	0.25	1.47	0.12	0.00E+00	-3.96E+01
cg27120999	14	64077803	HSPA2	heat shock 70kDa protein 2	0.50	0.34	1.46	0.16	7.00E-04	-7.24E+00
cg23616741	7	44071959	PGAM2	phosphoglycerate mutase 2 (muscle)	0.54	0.38	1.45	0.17	0.00E+00	-2.31E+01
cg02196655	2	10748215	NOL10	nucleolar protein 10	0.49	0.34	1.43	0.15	2.71E-02	-3.61E+00
cg12564453	16	55553341	CETP	cholesteryl ester transfer protein; plasma precurso	0.85	0.60	1.43	0.26	4.10E-03	-5.50E+00
cg27390220	3	45243527	RIS1	Ras-induced senescence 1	0.57	0.40	1.42	0.17	0.00E+00	-2.24E+01
cg16604218	1	45225192	EIF2B3	eukaryotic translation initiation factor 2B; subunit :	0.41	0.29	1.42	0.12	0.00E+00	-3.73E+01
cg13302154	12	14930699	MGP	matrix Gla protein	0.45	0.32	1.41	0.13	0.00E+00	-1.34E+01
cg25948180	11	34893357	PDHX	pyruvate dehydrogenase complex; component X	0.43	0.31	1.40	0.12	0.00E+00	-1.81E+01
cg27631817	6	10168924	OFCC1	MRDS1	0.36	0.25	1.40	0.10	0.00E+00	-3.06E+01
cg21096966	14	44502208	BTBD5	BTB (POZ) domain containing 5	0.68	0.49	1.40	0.19	0.00E+00	-1.03E+01
cg24107665	8	144444307	ZNF696	zinc finger protein 696	0.36	0.26	1.39	0.10	0.00E+00	-1.79E+01
cg18881501	17	21127969	MAP2K3	mitogen-activated protein kinase kinase 3 isoform	0.38	0.27	1.39	0.11	0.00E+00	-1.68E+01
cg09462576	1	226364496	MRPL55	mitochondrial ribosomal protein L55 isoform a	0.58	0.41	1.39	0.16	0.00E+00	-1.04E+01
cg06806711	11	59979867	MS4A1	membrane-spanning 4-domains; subfamily A; me	0.43	0.31	1.39	0.12	0.00E+00	-2.38E+01
cg20035459	16	3008708	CLDN6	claudin 6	0.58	0.42	1.38	0.16	0.00E+00	-3.09E+01
cg26372517	1	35811746	TFAP2E	transcription factor AP-2 epsilon (activating enhar	0.51	0.37	1.38	0.14	0.00E+00	-1.62E+01
cg11120551	1	145180620	CHD1L	chromodomain helicase DNA binding protein 1-lik	0.43	0.31	1.38	0.12	0.00E+00	-2.48E+01
cg16991589	13	24568047	PABPC3	poly(A) binding protein; cytoplasmic 3	0.54	0.39	1.37	0.14	3.72E+01	3.62E+00
cg08409225	12	54904004	OBFC2B	hypothetical protein LOC79035	0.47	0.34	1.37	0.13	0.00E+00	-3.75E+01
cg25133685	6	29121315	OR2W1	olfactory receptor; family 2; subfamily W; member	0.52	0.38	1.36	0.14	0.00E+00	-2.89E+01
cg08137040	7	126820931	LOC168850	hypothetical protein LOC168850	0.39	0.29	1.36	0.10	0.00E+00	-4.42E+01
cg20743744	4	1233849	CTBP1	C-terminal binding protein 1 isoform 1	0.43	0.31	1.35	0.11	0.00E+00	-2.99E+01
cg00839584	2	113258562	IL1A	interleukin 1; alpha proprotein	0.64	0.47	1.35	0.17	0.00E+00	-3.41E+01
cg26057752	7	44071460	PGAM2	phosphoglycerate mutase 2 (muscle)	0.61	0.45	1.35	0.16	0.00E+00	-2.88E+01
cg15352829	14	104462063	PLD4	phospholipase D family; member 4	0.41	0.30	1.35	0.10	0.00E+00	-1.08E+01
cg05670596	3	46423500	CCRL2	chemokine (C-C motif) receptor-like 2	0.39	0.29	1.34	0.10	0.00E+00	-1.85E+01
cg00846036	11	84311774	DLG2	chapsyn-110	0.50	0.37	1.34	0.13	0.00E+00	-3.26E+01
cg10669058	19	19509555	CILP2	cartilage intermediate layer protein 2	0.60	0.45	1.34	0.15	0.00E+00	-1.93E+01
cg24674703	11	60626536	CD5	CD5 antigen (p56-62)	0.54	0.41	1.34	0.14	0.00E+00	-2.62E+01
cg14920846	1	199884832	NAV1	neuron navigator 1	0.75	0.56	1.34	0.19	0.00E+00	-1.17E+01

cg13975369	7	129867789	TSGA14	testis specific; 14	0.47	0.35	1.32	0.11	0.00E+00	-3.33E+01
cg14460735	1	244795350	C1orf71	hypothetical protein LOC163882	0.50	0.38	1.32	0.12	0.00E+00	-3.82E+01
cg24019851	3	50289295	SEMA3B	semaphorin 3B isoform 2 precursor	0.68	0.52	1.31	0.16	0.00E+00	-1.32E+01
cg15284635	3	10833504	SLC6A11	solute carrier family 6 (neurotransmitter transporte	0.53	0.41	1.30	0.12	0.00E+00	-5.34E+01
cg24169822	7	27137519	HOXA4	homeobox protein A4	0.69	0.54	1.29	0.16	0.00E+00	-2.42E+01
cg26453588	22	41835965	BIK	BCL2-interacting killer	0.58	0.45	1.29	0.13	0.00E+00	-2.40E+01
cg12158019	1	40009903	OXCT2	3-oxoacid CoA transferase 2	0.67	0.52	1.29	0.15	0.00E+00	-1.66E+01
cg01399317	8	144882327	FLJ46072	hypothetical protein LOC286077	0.82	0.64	1.28	0.18	0.00E+00	-2.96E+01
cg19664945	2	53940847	GPR75	G protein-coupled receptor 75	0.61	0.78	0.79	-0.16	2.18E+11	2.61E+01
cg15095327	3	9919512	IL17RE	interleukin 17 receptor E isoform 3	0.42	0.54	0.78	-0.12	2.92E+09	2.18E+01
cg01459453	1	167865836	SELP	selectin P precursor	0.54	0.70	0.78	-0.16	2.00E+04	9.91E+00
cg17250929	1	151780434	S100A5	S100 calcium binding protein A5	0.36	0.47	0.77	-0.11	1.41E+07	1.65E+01
cg21929875	11	124127306	VSIG2	V-set and immunoglobulin domain containing 2	0.68	0.90	0.76	-0.22	1.34E+11	2.56E+01
cg04861271	14	22859371	PABPN1	poly(A) binding protein; nuclear 1	0.67	0.90	0.74	-0.23	1.23E+05	1.17E+01
cg03874127	6	109275828	ARMC2	armadillo repeat containing 2	0.40	0.55	0.73	-0.15	2.04E+06	1.45E+01
cg07220939	11	64115193	SLC22A12	urate anion exchanger 1 isoform a	0.29	0.41	0.71	-0.12	6.50E+09	2.26E+01
cg05181279	11	11939370	RIG	hypothetical protein LOC10530	0.51	0.71	0.71	-0.21	1.20E+08	1.86E+01
cg00579402	19	5789999	FUT6	fucosyltransferase 6 (alpha (1;3) fucosyltransferas	0.49	0.71	0.69	-0.22	3.42E+09	2.20E+01
cg22563815	15	76644004	CHRNA5	cholinergic receptor; nicotinic; alpha polypeptide 5	0.38	0.55	0.69	-0.17	5.55E+05	1.32E+01
cg18382305	18	29275063	C18orf34	hypothetical protein LOC374864	0.31	0.45	0.69	-0.14	8.12E+07	1.82E+01
cg11787522	15	72282162	STRA6	stimulated by retinoic acid gene 6 homolog	0.23	0.33	0.68	-0.11	1.09E+07	1.62E+01
cg01919208	3	49145500	LAMB2	laminin; beta 2 precursor	0.34	0.50	0.68	-0.16	6.01E+08	2.02E+01
cg26581729	9	139059613	NPDC1	neural proliferation; differentiation and control; 1	0.31	0.45	0.68	-0.15	1.02E+10	2.30E+01
cg08471713	17	39094419	MEOX1	mesenchyme homeo box 1 isoform 2	0.40	0.59	0.68	-0.19	1.06E+03	6.96E+00
cg20857253	21	32879161	TCP10L	T-complex 10A-2	0.26	0.39	0.67	-0.13	4.07E+08	1.98E+01
cg23732182	21	43722518	C21orf84	hypothetical protein LOC114038	0.39	0.58	0.67	-0.19	3.78E+07	1.74E+01
cg09809672	1	234624305	EDARADD	EDAR-associated death domain isoform B	0.32	0.48	0.67	-0.16	3.80E+11	2.67E+01
cg26743024	17	62390744	CACNG4	voltage-dependent calcium channel gamma-4 sub	0.30	0.45	0.67	-0.15	7.44E+04	1.12E+01
cg02082342	11	124127583	VSIG2	V-set and immunoglobulin domain containing 2	0.50	0.76	0.66	-0.26	3.89E+10	2.44E+01
cg03543593	6	32184395	TNXB	tenascin XB isoform 2	0.30	0.46	0.65	-0.16	3.83E+07	1.75E+01
cg17983064	5	131424256	IL3	interleukin 3 precursor	0.44	0.68	0.64	-0.25	2.59E+07	1.71E+01
cg01479232	20	696992	C20orf54	hypothetical protein LOC113278	0.39	0.64	0.62	-0.24	5.22E+08	2.01E+01
cg21918500	1	245402094	ZNF124	zinc finger protein 124	0.16	0.27	0.59	-0.11	2.68E+06	1.48E+01
cg07732037	12	122273780	MPHOSPH9	M-phase phosphoprotein 9	0.16	0.28	0.57	-0.12	1.63E+07	1.66E+01
cg09554443	1	165754386	CD3Z_CD247	T-cell receptor zeta chain isoform 2 precursor	0.14	0.26	0.55	-0.12	1.03E+06	1.38E+01
cg12024906	19	42517519	HKR1	GLI-Kruppel family member HKR1	0.20	0.37	0.55	-0.16	9.24E+09	2.29E+01
cg13185177	3	195601174	GP5	glycoprotein V (platelet)	0.30	0.55	0.55	-0.25	1.31E+08	1.87E+01
cg04972979	20	697148	C20orf54	hypothetical protein LOC113278	0.16	0.31	0.50	-0.16	2.66E+11	2.63E+01
cg09871315	7	27109207	HOXA2	homeobox A2	0.10	0.20	0.48	-0.11	9.46E+18	4.37E+01
