# Supplementary material for: Altered DNA Methylation in Leukocytes with Trisomy 21
Source: PLoS Genet. 2010 Nov 18;6(11):e1001212. doi: 10.1371/journal.pgen.1001212 (PMC2987931; doi:10.1371/journal.pgen.1001212)
Supplement: Table S3 — Stability of DNA methylation over time in adults with DS. (0.05 MB PDF) [file pgen.1001212.s010.pdf]

Gene	median value controls	median value DS	DS Study ID number	Age	Percent methylation (MS-Pyroseq)
TCF7	44	24.8	33	64.4	23.1
			33	67.8	22.5
			33	70.9	27.0
			691	62.6	21.1
			691	65.4	21.1
			691	68.7	26.0
			162	47	24.9
			162	50	27.2
			162	53	20.0
			162	54	23.0
			178	45	33.5
			178	50	32.0
			178	52	26.3
			178	54	22.4
			564	54	20.1
			564	57.2	20.0
			122	78	36.1
			122	79.7	36.9
TMEM131	39.2	9.2	33	64.4	8.5
			33	67.8	9.6
			33	70.9	13.2
			162	47	14.4
			162	50	18.6
			162	53	13.7
			162	54	20.9
			178	45	1.8
			178	50	4.6
			178	52	6.1
			178	54	3.3
			691	62.6	6.3
			691	65.4	6.1
			691	68.7	5.0
			564	54	8.3
			564	57.2	5.4
			122	78	4.5
			122	79.7	6.8
