# Supplementary material for: Altered DNA Methylation in Leukocytes with Trisomy 21
Source: PLoS Genet. 2010 Nov 18;6(11):e1001212. doi: 10.1371/journal.pgen.1001212 (PMC2987931; doi:10.1371/journal.pgen.1001212)
Supplement: Table S4 — Methylation values in cases of DS with mosaicism. (0.06 MB PDF) [file pgen.1001212.s011.pdf]

Lab ID	DS study ID	Age	Karyotype	cells with normal diploid karyotype	Gene	Percent Methylation (MS-Pyroseq)	median value controls	median value DS
ds146	484	51	47,XX,+21 (22), 46,XX (78)	78%	*TCF7*	*44.5*	44	24.8
ds690	1718	43	47,XX,+21 (16), 46,XX (34)	68%		37.9		
ds701	569	56	47,XY,+21 (39), 46XY (11)	22%		24.5		
ds329	122	80	47,XX,+21 (43), 46XX (5)	10%		36.9		
ds094	526	62	47,XY,+21 (46), 46XY (4)	8%		28.3		
ds146	484	51	47,XX,+21 (22), 46,XX (78)	78%	*TMEM131*	36.6	39.2	9.2
ds690	1718	43	47,XX,+21 (16), 46,XX (34)	68%		20.7		
ds701	569	56	47,XY,+21 (39), 46XY (11)	22%		11.7		
ds329	122	80	47,XX,+21 (43), 46XX (5)	10%		6.8		
ds094	526	62	47,XY,+21 (46), 46XY (4)	8%		10.5		
ds146	484	51	47,XX,+21 (22), 46,XX (78)	78%	*SH3BP2*	17.9	21.7	31.5
ds690	1718	43	47,XX,+21 (16), 46,XX (34)	68%		18.2		
ds701	569	56	47,XY,+21 (39), 46XY (11)	22%		19.2		
ds329	122	80	47,XX,+21 (43), 46XX (5)	10%		39.4		
ds094	526	62	47,XY,+21 (46), 46XY (4)	8%		41.5		
