# Supplementary material for: Altered DNA Methylation in Leukocytes with Trisomy 21
Source: PLoS Genet. 2010 Nov 18;6(11):e1001212. doi: 10.1371/journal.pgen.1001212 (PMC2987931; doi:10.1371/journal.pgen.1001212)
Supplement: Table S5 — Complete blood counts in 4 adults with DS. The normal ranges for Columbia University Medical Center are indicated. (0.06 MB PDF) [file pgen.1001212.s012.pdf]

Case ID	DS 700	DS 701	DS 702	DS 703	Mean % Methyl all DS PBL	Mean % Methyl all control PBL
***TMEM131* MI**	15.3	11.7	13.7	7.6	10.9	41.1
***TCF7* MI**	27.3	24.5	32.2	33.1	26.5	44
***SH3BP2* MI**	46	19.21	31.53	41.5	34.5	23.6
					**Normal Range (%)**	
**WBC, number**	7.76	5.56	3.59	4.69	3.54-9.06	
**Neutrophils, (%)**	73.5	56.9	45	54	40-70%	
**Lymphocytes, (%)**	18.4	29	43.5	35	20-50%	
**Monocytes, (%)**	6.4	11.9	10.3	10.2	4-8%	
