# Supplementary material for: Altered DNA Methylation in Leukocytes with Trisomy 21
Source: PLoS Genet. 2010 Nov 18;6(11):e1001212. doi: 10.1371/journal.pgen.1001212 (PMC2987931; doi:10.1371/journal.pgen.1001212)
Supplement: Table S6 — Differentially methylated loci from the Illumina Infinium screen using DNA from purified T-cells. Loci with increased methylation in DS are in red and loci with decreased methylation in DS are in blue. Statistical criteria were ANOVA p<.01; >1.3 fold difference in methylation index and >0.15 absolute difference in methylation index between DS T-cells and normal T-cells. One further example, the CD3Z gene, was also differentially methylated both in total PBL and in T-cells, as indicated by the MS-Pyroseq data (Figure S5), but as the methylation values were all in a very low range (2 - 14% methylation) the MS-Pyroseq was more senstive than the Infinium assay for detecting the difference. One gene, ALX4, which is queried by the Infinium BeadChip at two different CpG dinucleotides separated by 5.5 kb, showed opposite changes in fractional methylation at these two positions. (0.07 MB PDF) [file pgen.1001212.s013.pdf]

probe set	Chrom	Position	Gene Name	av DS T-cells	av control T-cells	av DS / av control	av DS - av control
cg14859460	5	178354850	GRM6	0.46	0.11	4.33	0.35
cg07846220	18	7107680	LAMA1	0.16	0.04	4.31	0.12
cg24674703	11	60626536	CD5	0.14	0.03	4.31	0.11
cg22836229	8	49810270	EFCAB1	0.18	0.05	3.40	0.13
cg10756887	22	22435692	C22orf15	0.29	0.09	3.35	0.20
cg27009703	7	27171419	HOXA9	0.22	0.07	3.21	0.15
cg09688546	4	158216813	GLRB	0.17	0.05	3.21	0.12
cg15731815	1	6191847	C1orf188	0.22	0.07	3.19	0.15
cg20727114	19	54885932	CPT1C	0.38	0.13	2.95	0.25
cg23833452	6	146906445	RAB32	0.18	0.06	2.94	0.12
cg20318748	20	25553178	NANP	0.23	0.08	2.83	0.15
cg16248277	10	103526053	FGF8	0.18	0.06	2.83	0.11
cg26504021	5	2805882	IRX2	0.22	0.08	2.80	0.14
cg14917512	19	3045685	GNA11	0.30	0.11	2.74	0.19
cg22791453	9	132309720	ASS	0.22	0.08	2.73	0.14
cg12080675	5	179267829	KIAA0676	0.23	0.08	2.73	0.14
cg08822227	4	2790266	SH3BP2	0.55	0.21	2.69	0.35
cg02613386	17	6620257	FBXO39	0.28	0.11	2.62	0.17
cg04747322	5	121675207	SNCAIP	0.25	0.10	2.55	0.15
cg08797106	6	26128987	HIST1H3A	0.17	0.07	2.54	0.10
cg02497700	1	242279981	ZNF238	0.23	0.09	2.54	0.14
cg22285621	11	66827898	SSH3	0.24	0.10	2.53	0.15
cg26985289	1	9807209	CLSTN1	0.21	0.09	2.47	0.13
cg02988947	17	59132545	LIMD2	0.18	0.07	2.46	0.11
cg21243096	1	38284144	POU3F1	0.18	0.07	2.46	0.11
cg24794531	3	143926527	TRPC1	0.17	0.07	2.45	0.10
cg25983380	20	56898834	GNAS	0.33	0.14	2.44	0.20
cg21561173	21	14274634	C21orf81	0.32	0.13	2.38	0.18
cg10313673	19	19510144	CILP2	0.21	0.09	2.35	0.12
cg02600394	4	47830991	TXK	0.26	0.11	2.35	0.15
cg14972143	4	100070026	EIF4E	0.25	0.11	2.34	0.14
cg05337441	2	21120073	APOB	0.21	0.09	2.33	0.12
cg05222924	11	32407062	WT1	0.27	0.12	2.32	0.15
cg25432696	4	74921592	CXCL6	0.21	0.09	2.29	0.12
cg02250594	18	53254351	ONECUT2	0.27	0.12	2.27	0.15
cg13929328	10	129425888	FLJ46831	0.27	0.12	2.27	0.15
cg16632715	2	176680359	HOXD11	0.30	0.13	2.25	0.17
cg27118825	19	51010588	RSHL1	0.30	0.14	2.22	0.17
cg09088834	20	25513460	KIAA0980	0.34	0.16	2.18	0.18
cg00365193	6	2944394	NQO2	0.23	0.11	2.12	0.12
cg20029201	11	118287023	BCL9L	0.47	0.22	2.12	0.25
cg09107315	1	78284076	GIPC2	0.30	0.14	2.11	0.16
cg24989962	14	51804036	PTGDR	0.24	0.12	2.10	0.13
cg04887278	6	83960646	RWDD2	0.28	0.14	2.10	0.15
cg07991621	4	2790277	SH3BP2	0.54	0.26	2.10	0.28
cg16232126	2	107969437	SLC5A7	0.22	0.11	2.08	0.11

cg24256211	14	54104965	SAMD4A	0.41	0.20	2.08	0.21
cg22402007	9	86472643	NTRK2	0.19	0.09	2.07	0.10
cg19450025	16	30113574	SULT1A3	0.29	0.14	2.06	0.15
cg01806928	11	7229380	SYT9	0.34	0.17	2.05	0.17
cg10056627	6	43036751	GNMT	0.21	0.10	2.05	0.11
cg25711779	2	56003329	EFEMP1	0.22	0.11	2.05	0.11
cg04797323	12	92491109	SOCS2	0.25	0.12	2.04	0.13
cg08832227	12	4890954	KCNA1	0.51	0.25	2.03	0.26
cg10660256	5	78443439	BHMT	0.22	0.11	2.03	0.11
cg15489294	5	115325752	FLJ90650	0.29	0.14	2.02	0.15
cg25186143	17	581814	FAM57A	0.32	0.16	2.02	0.16
cg15439196	10	104666897	CNNM2	0.32	0.16	1.98	0.16
cg02498063	12	118256960	CCDC60	0.73	0.37	1.97	0.36
cg11507178	11	118286725	BCL9L	0.38	0.19	1.97	0.19
cg14348532	11	14950138	CALCA	0.28	0.14	1.96	0.14
cg27491887	11	2511159	KCNQ1	0.27	0.14	1.96	0.13
cg13870866	7	35259655	TBX20	0.23	0.12	1.96	0.11
cg20430816	4	77447378	GENX-3414	0.23	0.12	1.96	0.11
cg09963123	4	38341049	KLF3	0.29	0.15	1.95	0.14
cg22892110	8	144870619	MAPK15	0.33	0.17	1.94	0.16
cg11027330	16	704795	METRN	0.32	0.16	1.94	0.15
cg09626984	8	11603505	GATA4	0.25	0.13	1.93	0.12
cg13599477	10	5478485	NET1	0.28	0.15	1.93	0.14
cg10557828	3	147362332	PLOD2	0.44	0.23	1.92	0.21
cg23616741	7	44071959	PGAM2	0.59	0.31	1.91	0.28
cg22485810	17	26272851	CENTA2	0.36	0.19	1.90	0.17
cg15703690	7	22733517	IL6	0.28	0.15	1.90	0.13
cg03811478	3	138966386	SOX14	0.28	0.15	1.90	0.13
cg18771300	14	62741490	RHOJ	0.71	0.38	1.89	0.34
cg25141674	6	72187410	C6orf155	0.23	0.12	1.89	0.11
cg07638935	10	102811417	KAZALD1	0.21	0.11	1.88	0.10
cg19601035	12	46685407	COL2A1	0.23	0.12	1.88	0.11
cg24396745	15	71447667	HCN4	0.23	0.12	1.88	0.11
cg10737521	5	179267849	KIAA0676	0.23	0.12	1.85	0.10
cg06101212	7	72822156	CLDN3	0.29	0.16	1.85	0.13
cg23591869	11	110675858	FLJ45803	0.24	0.13	1.85	0.11
cg24715735	19	40223243	HPN	0.37	0.20	1.84	0.17
cg13035743	6	32227663	PRRT1	0.28	0.15	1.84	0.13
cg26133068	22	22529008	SLC2A11	0.35	0.19	1.83	0.16
cg09715672	10	88718258	C10orf116	0.25	0.14	1.83	0.11
cg01546046	14	30564600	AP4S1	0.26	0.14	1.81	0.12
cg19764436	22	21743260	GNAZ	0.25	0.14	1.81	0.11
cg17272843	11	77411928	KCTD14	0.29	0.16	1.81	0.13
cg08569678	8	143778399	LY6K	0.37	0.21	1.80	0.17
cg20345446	11	3143380	OSBPL5	0.31	0.17	1.79	0.14
cg04452713	6	56815646	DST	0.28	0.16	1.79	0.12
cg26912636	20	55719797	TMEPAI	0.47	0.27	1.76	0.20
cg21517055	19	13877717	MGC11271	0.41	0.23	1.76	0.18

cg05380982	21	38210248	KCNJ6	0.25	0.14	1.75	0.11
cg09837977	7	110518437	LRRN3	0.74	0.42	1.75	0.32
cg04598121	8	57521059	PENK	0.29	0.17	1.75	0.12
cg04884908	2	72228348	CYP26B1	0.25	0.15	1.75	0.11
cg26385222	7	150128010	HCA112	0.70	0.40	1.75	0.30
cg20483374	11	118716856	C1QTNF5	0.43	0.25	1.74	0.18
cg02919422	8	55533097	SOX17	0.31	0.18	1.74	0.13
cg11680741	17	10266615	MYH8	0.66	0.38	1.74	0.28
cg21917349	15	27001152	APBA2	0.36	0.21	1.72	0.15
cg00643392	19	12807856	RTBDN	0.35	0.21	1.72	0.15
cg12457773	6	24234476	VMP	0.25	0.15	1.71	0.10
cg25689955	11	47558276	KBTBD4	0.78	0.46	1.70	0.32
cg12762799	12	8584809	CLEC4E	0.48	0.28	1.70	0.20
cg11797994	11	44287307	ALX4	0.40	0.24	1.70	0.17
cg04675937	9	21995419	CDKN2B	0.31	0.18	1.69	0.13
cg09305478	16	631989	MGC15416	0.26	0.15	1.68	0.10
cg09230173	9	70161179	PGM5	0.26	0.15	1.68	0.10
cg09188980	11	14949954	CALCA	0.33	0.20	1.68	0.13
cg00660989	14	44502266	BTBD5	0.70	0.42	1.67	0.28
cg26256793	1	103347057	COL11A1	0.27	0.17	1.66	0.11
cg23555120	12	105057993	NUAK1	0.31	0.19	1.65	0.12
cg19096540	5	133776090	MGC13017	0.37	0.22	1.65	0.14
cg11004890	20	3166500	SLC4A11	0.72	0.44	1.65	0.28
cg27360098	7	73080416	ELN	0.52	0.32	1.63	0.20
cg05067286	11	129191575	TMEM45B	0.43	0.26	1.63	0.17
cg13749822	4	145786113	HHIP	0.56	0.35	1.62	0.22
cg25358289	5	139992912	CD14	0.33	0.21	1.61	0.13
cg13817266	1	159761026	HSPA6	0.49	0.30	1.60	0.18
cg09606564	17	19230946	MFAP4	0.38	0.24	1.57	0.14
cg18087477	1	115199120	SYCP1	0.59	0.38	1.53	0.20
cg03733371	3	186753222	LIPH	0.35	0.58	0.60	-0.23
cg05200628	1	158948384	CD48	0.15	0.27	0.55	-0.12
cg20286074	17	39350062	APR-2	0.24	0.44	0.54	-0.20
cg04340502	6	52883168	GSTA3	0.15	0.29	0.52	-0.14
cg00061629	11	44281834	ALX4	0.12	0.23	0.52	-0.11
cg05995267	16	29730030	PRRT2	0.12	0.24	0.51	-0.12
cg16310717	12	4253880	CCND2	0.10	0.21	0.48	-0.11
cg12832565	1	144427029	CD160	0.12	0.27	0.47	-0.14
cg26954174	16	49288314	NOD2_CARD15	0.15	0.34	0.44	-0.19
cg23732024	8	75066355	LY96	0.10	0.23	0.43	-0.13
cg16363586	19	17377329	BST2	0.08	0.19	0.41	-0.11
cg02057157	4	6768582	CNO	0.07	0.19	0.38	-0.12
cg08471713	17	39094419	MEOX1	0.25	0.67	0.38	-0.42
cg01253545	22	29886314	RNF185	0.07	0.20	0.36	-0.13
cg27400772	19	43406904	DPF1	0.06	0.18	0.35	-0.12
cg26131019	3	66633425	LRIG1	0.04	0.14	0.25	-0.11
