# Supplementary material for: Altered DNA Methylation in Leukocytes with Trisomy 21
Source: PLoS Genet. 2010 Nov 18;6(11):e1001212. doi: 10.1371/journal.pgen.1001212 (PMC2987931; doi:10.1371/journal.pgen.1001212)
Supplement: Table S7 — PCR primers used in this study. (0.05 MB PDF) [file pgen.1001212.s014.pdf]

**Bisulfite Sequencing_COBRA_MS-Pyroseq**	**SNP ID or Infinium cg ID**	**Change in DS vs. normal PBL**	**Differentially methylated CpG sites**	**CpG sites Pyroseq**	**CpG sites**	**Temp**	**COBRA Enzyme(s)**	**Upstream**	**Downstream**		**size (bp)**
FAM62C	rs1720830	GOM	CpG pos #1-10	10	14	57→48	*Bst* UI	TTGATTATTAAAGGGTTAGAAATTATGG	ATAAAACTTAAACCTAACCCCCAAC	CONVERTED	290
								CTGATCATTAAAGGGTTAGAAACCATGG	GTAAAGCTTGGACCTAGCCCCCAGC	UNCONVERTED	
TCF7 upstream region	rs756699	LOM	CpG pos #1-8	8	8	57→48	*Hpy* CH4IV	TTTTGTTTTGAGAGGATGTTTTATG	TTCTCATAACCTAAACCCACTTAATC	CONVERTED	330
								TTTTGCCCTGAGAGGATGCTCCATG	TTCTCATGGCCTGAGCCCACTTAATC	UNCONVERTED	
TCF7 CGI	NA	GOM	CpG pos #1-17	17	17	60→51	NA	AGGGGAGTTGTTGTTGATTGTAT	AACCCTAACCTAACTAACCCTCTAAC	CONVERTED	216
								AGGGGAGCTGCTGTTGACTGCAT	GACCCTGGCCTGACTAACCCTCTAAC	UNCONVERTED	
TMEM131 index	rs6760008	LOM	CpG pos #9-21	13	21	60→51	*Bst* UI/*Hpy* CH4IV	GGTAGGAAATGTAGAGGGTAGTTAT	CCTAACCACACAAACAAAATACAAA	CONVERTED	339
								GGCAGGAAATGCAGAGGGCAGCCAC	CCTGACCACACAGACAGGATGCAGG	UNCONVERTED	
TMEM131 CGI	NA	none	NA		34	57→48	NA	GAAGGAGGGAGGTAGGTTTAGG	TCAAAAAAACACCATCAAAAACTAA	CONVERTED	360
								GAAGGAGGGAGGCAGGCCCAGGTC	TCAAGGAAGCACCATCAAGAACTGAG	UNCONVERTED	
SH3BP2 CGI	cg08822227	GOM	CpG pos #8-17	17	17	56→47	*Bst* UI	TAGATTTTTGGTTTTTTGGAGAATT	ACACCTACCAATCCACACAACTTA	CONVERTED	258
								CAGACCCCTGGCTCCTTGGAGAATC	ACACCTGCCAGTCCACACAGCTTG	UNCONVERTED	
EIF4E	cg14972143	GOM	CpG pos #3-16	14	16	57→48	*Hpy* CH4IV	GTTTAGTTAATGTTGGTGGTTATT	CTAACATCTAAACTTAAACTATTTCC	CONVERTED	246
								GCTCAGCTAATGCTGGTGGTCATT	CTGGCATCTGGACTTAGGCTATTTCC	UNCONVERTED	
SUMO3	cg21053323	GOM	CpG pos #7-13	11	11	N/A	N/A	TTTATAGGGGTTAGGTTTTTTAGAG	TCTCCTAAATCATTACTAATTAAAATC	CONVERTED	149
								CTCACAGGGGCCAGGCCCCTCAGAG	TCTCCTGAGTCATTGCTGGTTGGGGTC	UNCONVERTED	
PLD6/LOC201164	cg05590257	GOM	CpG pos #1-16	16	22	57→48	*Bst* UI	TTTTTTTTAAGTGTTTATTTTTG	CCAACCTAAATCTCTACCTATTC	CONVERTED	235
								CCTCCCCTAAGTGTCCACCTTTG	CCAGCCTGGATCTCTGCCTGTTC	UNCONVERTED	
CD3Z	cg09554443	LOM	CpG pos #9-11	3	11	63→54	*Bst* UI/*Hpy* 188III	GGGTAGGATTTGAAGGAGATTTTAG	CAATCCTCCACTTCCTAAAAAAATA	CONVERTED	300
								GGGCAGGATTTGAAGGAGACCCCAG	CAGTCCTCCACTTCCTGGGGAGGTA	UNCONVERTED	
CPT1B	cg00983520	GOM	CpG pos #1-10	10	9	60→51	NA	AGGAATTTGATATTTATTTTTAAAT	ACTCCCAAACCTTTAAACAACCT	CONVERTED	139
								AGGAACCTGACACCTACTCCCAAAT	GCTCCCAGACCTTTGAGCAACCT	UNCONVERTED	
NOD2	cg26954174	LOM	N/A	N/A	6	61→52	NA	GATGTAGTTGTTGGGAGGATAGAGT	CCCCACTAAAAACATAAAACACATT	CONVERTED	207
								GATGTAGCTGCTGGGAGGACAGAGC	CCCCACTGGGGACATGAGACACATT	UNCONVERTED	
**Q-PCR**	**Exons**	**Primer Set**						**Upstream**	**Downstream**		
TMEM131 long isoform	Ex 19-20	F5/R5						CAATAATTTCAAGCCTGCCAGA	CGGTATAGCCTGAAGCTAATGTTACCG		76
TMEM131 short isoform	5'UTR to Ex3	F3/R3						GACATGAGGCTGAGCGATG	GAACGAATTGAGGGGAGTGA		422
TCF7	Ex 2-4 long isoforms	F2/R2 (KK)						TACTCCGCCTTCAATCTGCT	TGCCTGAGGTCAGGGAGTAG		241
NOD2	Ex 3-4	F4/R4						GCCCTGCCTTTGGAAGCTGC	CGCTTCTCCATCATAGGTACTGAGGAAGCG		97
NPDC1	Ex 7-8	F2/R2						CGTTGTACCACTACCAGCACCAACG	GCTCCTTGGGTGGCTCTTTATG		68
HPRT	ex 4-6	F3/R3						GACCAGTCAACAGGGGACAT	CCTGACCAAGGAAAGCAAAG		132
